# Germinal center cytokine driven epigenetic control of Epstein-Barr virus latency gene expression

**DOI:** 10.1371/journal.ppat.1011939

**Published:** 2024-04-29

**Authors:** Yifei Liao, Jinjie Yan, Nina R. Beri, Lisa Giulino-Roth, Ethel Cesarman, Benjamin E. Gewurz

**Affiliations:** 1 Division of Infectious Disease, Department of Medicine, Brigham and Women’s Hospital, Harvard Medical School, Boston, Massachusetts, United States of America; 2 Center for Integrated Solutions to Infectious Diseases, Broad Institute of Harvard and MIT, Cambridge, Massachusetts, United States of America; 3 Department of Microbiology, Harvard Medical School, Boston, Massachusetts, United States of America; 4 Sun Yat-Sen University Cancer Center, State Key Laboratory of Oncology in South China, Guangzhou, China; 5 Weill Cornell Medical College, New York, New York, United States of America; 6 Harvard Program in Virology, Harvard Medical School, Boston, Massachusetts, United States of America; University of Florida, UNITED STATES

## Abstract

Epstein-Barr virus (EBV) persistently infects 95% of adults worldwide and is associated with multiple human lymphomas that express characteristic EBV latency programs used by the virus to navigate the B-cell compartment. Upon primary infection, the EBV latency III program, comprised of six Epstein-Barr Nuclear Antigens (EBNA) and two Latent Membrane Protein (LMP) antigens, drives infected B-cells into germinal center (GC). By incompletely understood mechanisms, GC microenvironmental cues trigger the EBV genome to switch to the latency II program, comprised of EBNA1, LMP1 and LMP2A and observed in GC-derived Hodgkin lymphoma. To gain insights into pathways and epigenetic mechanisms that control EBV latency reprogramming as EBV-infected B-cells encounter microenvironmental cues, we characterized GC cytokine effects on EBV latency protein expression and on the EBV epigenome. We confirmed and extended prior studies highlighting GC cytokine effects in support of the latency II transition. The T-follicular helper cytokine interleukin 21 (IL-21), which is a major regulator of GC responses, and to a lesser extent IL-4 and IL-10, hyper-induced LMP1 expression, while repressing EBNA expression. However, follicular dendritic cell cytokines including IL-15 and IL-27 downmodulate EBNA but not LMP1 expression. CRISPR editing highlighted that STAT3 and STAT5 were necessary for cytokine mediated EBNA silencing via epigenetic effects at the EBV genomic C promoter. By contrast, STAT3 was instead necessary for LMP1 promoter epigenetic remodeling, including gain of activating histone chromatin marks and loss of repressive polycomb repressive complex silencing marks. Thus, EBV has evolved to coopt STAT signaling to oppositely regulate the epigenetic status of key viral genomic promoters in response to GC cytokine cues.

## Author summary

A longstanding question has remained how Epstein-Barr virus (EBV) epigenetically switches between latency programs as it navigates the B-cell compartment. EBV uses its latency III program to stimulate newly infected B cell growth and then trafficking into secondary lymphoid tissue germinal centers (GCs). In latency III, the viral C promoter stimulates expression of six Epstein-Barr nuclear antigens (EBNA) that in turn induce two latent membrane proteins (LMP). However, knowledge has remained incomplete about how GC microenvironmental cues trigger switching to latency II, where only one EBNA and two LMP are expressed, a program observed in Hodgkin lymphoma. Building on prior evidence that GC cytokines are a major cue, we systematically tested effects of cytokines secreted by GC-resident T follicular helper and follicular dendritic cells on EBV latency gene expression and on epigenetic remodeling of their promoters. This highlighted that a range of GC cytokines repress latency III EBNA, while only several support LMP1 expression, major events in the transition between the latency III and II programs. We identified key downstream roles of JAK/STAT signaling in relaying cytokine signals to the EBV epigenome, including obligatory STAT3 and 5 roles in rewiring of C and LMP promoter histone epigenetic marks.

## Introduction

Epstein-Barr virus (EBV) persistently infects >95% of adults worldwide. Although typically benign, EBV nonetheless contributes to approximately 1.5% of all human cancers [1]. These include endemic Burkitt lymphoma (BL), Hodgkin lymphoma, natural killer/T cell lymphoma, post-transplant lymphoproliferative disease (PTLD), primary central nervous system lymphoma and diffuse large B-cell lymphoma, which typically arise from the germinal center (GC) [1–3]. EBV is also highly associated with multiple sclerosis [4,5]. According to the EBV GC model, EBV uses distinct combinations of latent membrane proteins (LMP) and Epstein-Barr nuclear antigens (EBNA) to expand the pool of infected B-cells, navigate the B-cell compartment and promote infected cell differentiation into memory B-cells, the reservoir for lifelong infection [6]. Across these latency programs, ~80 viral lytic antigens are largely silenced by epigenetic mechanisms.

The EBV genome is epigenetically programmed upon B cell infection [7–10]. While EBV genomic DNA is epigenetically naïve in viral particles, it is rapidly chromatinized as incoming viral genomes reach the infected cell nucleus [7,11]. Histone epigenetic marks, DNA methylation and three dimensional EBV genomic architecture then serve as major regulators of EBV gene expression. Much remains to be learned about host cell transcription factors and their upstream pathways in control of EBV epigenomic programming. The viral W promoter (Wp) drives an initial burst of EBNA expression, in particular EBNA2 and EBNA-LP, which highly upregulate MYC and other key B-cell targets [12–19]. Infected cells then transition to the latency IIb program, in which the EBV genomic C promoter (Cp) drives expression of a transcript encoding EBNAs 1, 2, 3A, 3B, 3C and LP, whose messages are subsequently spliced. Shortly thereafter, EBNA2 activates the latent membrane promoters, driving expression also of LMP1 and LMP2A, culminating in the latency III program [3]. If left unchecked, the transforming latency III program converts B-cells into immortalized lymphoblastoid cell lines (LCL), a key model for PTLD and AIDS-associated immunoblastic lymphomas [1,9,20].

Latency III drives cells into GC, where immune pressure together with incompletely understood mechanisms are believed to drive the transition to the EBV latency II program, comprised of EBNA1, LMP1 and 2A [9]. EBNA1 expression is driven by the viral genome Q promoter (Qp) in latency II. Much remains to be understood about the precise GC signals and their downstream epigenetic mechanisms that culminate in Cp silencing, while instead supporting LMP expression in the absence of EBNA2 transcription activation. Upon memory B-cell differentiation, epigenetic mechanisms likely including DNA methylation and polycomb repressor complex 1 silence the LMP promoters to enable progression latency I program, where EBNA1 is the only EBV-encoded protein expressed [8,21].

The GC is a dynamic secondary lymphoid tissue microstructure, where T follicular helper (Tfh) and follicular dendritic cells (FDC) together with antigens drive B-cell responses [22,23]. Tfh cytokines, including IL-2, 4, 10, and 21, together with the FDC derived cytokines IL-6, 15 and 27, are critical for GC establishment and maintenance, as well as for GC B-cell fate [23–26]. Cytokines bind to plasma membrane B-cell receptors to activate Janus kinase (JAK) or Tyrosine kinase 2 (TYK2), which phosphorylate specific signal transducer and activator of transcription (STAT) family proteins. Phosphorylation drives STAT dimerization via reciprocal SH2 domain—phosphotyrosine interactions and nuclear translocation to enable target gene regulation (S1A Fig) [27–29]. IL-21 decreases EBNA2 expression in latency III B cells [30,31], suggesting a potential GC cytokine role in driving the transition from latency III to II. Moreover, IL-4, 10, and 21 each de-repress LMP1 expression in newly infected cells and in latency I Burkitt and natural killer (NK) lymphoma cells [30–36], further suggesting roles in support of latency II. IL-15 also drives NK and T-cell responses against EBV transformed peripheral blood B-cells [37,38], potentially suggesting that it may enhance immune pressure against latency III B-cells within the GC. However, much remains to be learned about the mechanisms by which cytokines secreted by Tfh and FDC alter the EBV epigenome to repress EBNA but instead support LMP expression.

To gain insights into mechanisms by which GC cytokines alter EBV latency gene expression and the viral epigenome, we systematically screened effects of Tfh and FDC cytokines on EBV latency gene expression. Tfh cytokines, including IL-4, 10 and 21, each upregulated LMP1 but downregulated EBNA2 and 3 levels in B cells with latency III. By contrast, the key FDC cytokine IL-15 diminished Cp driven EBNA expression but did not significantly alter LMP1 levels. CRISPR analysis identified that STAT3 and to a lesser extent STAT5 were critical for these cytokine effects on EBNA and LMP1 expression. Taken together, our results highlight GC cytokines driven STAT3 and 5 remodeling of the EBV epigenome to support the latency III to latency II program transition.

## Results

### GC cytokines support the latency II transition

**Fig 1:**
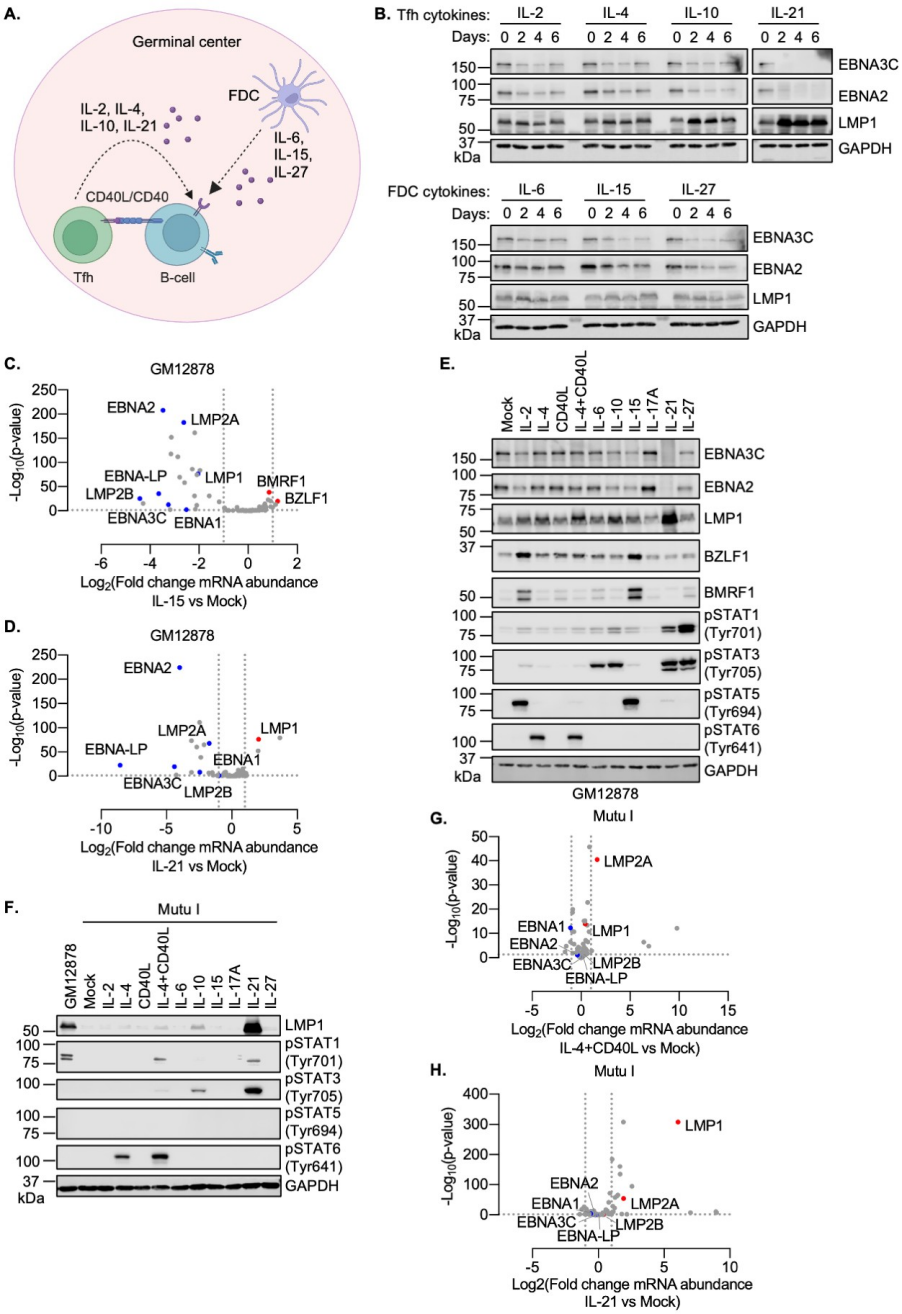
GC cytokines support the transition to EBV latency II. (**A**) GC schematic, illustrating key T follicular helper cell (Tfh) and follicular dendritic cell (FDC) secreted cytokines and CD40 ligand (CD40L) that signal to GC B-cells. (BioRender was used to create the schematic models.) (**B**) Immunoblot analysis of whole cell lysates (WCL) from GM12878 treated with the indicated cytokines for two, four, or six days. (**C**) Volcano plots of EBV gene expression from n = 3 replicates of GM12878 stimulated by IL-15 vs. mock-stimulated for 6 days. (**D**) Volcano plots of EBV gene expression from n = 3 replicates of GM12878 stimulated by IL-21 vs. mock-stimulated for 6 days. (**E**) Immunoblot analysis of WCL from GM12878 cells treated with the indicated cytokines for six days. (**F**) Immunoblot analysis of WCL from Mutu I cells treated with the indicated cytokine for one day or from GM12878 for comparison. (**G**) Volcano plot of EBV gene expression from n = 3 replicates of Mutu I stimulated by IL-4+CD40L vs. mock-stimulated for one day. (**H**) Volcano plot of EBV gene expression from n = 3 replicates of Mutu I stimulated by IL-21 vs. mock-stimulated for one day. All cytokines were used at 100 ng/ml and 50 ng/ml in GM12878 vs Mutu I, respectively, and were refreshed every two days. Immunoblots are representative of n = 3 replicates.

To systematically characterize GC cytokine effects on EBV latency gene expression, we incubated the LCL GM12878 with a panel of Tfh-derived cytokines, IL-2, IL-4, IL-10 or IL-21. In parallel, we incubated GM12878 with the FDC-derived cytokines IL-6, IL-15 or IL-27 for 0, 2, 4 or 6 days (Figs 1A and S1A). While it is not known how long EBV+ B-cells reside within the GC, it is likely that they remain present for at least several days, in order to proliferate and differentiate into memory B cells, and GC structures themselves persist for weeks to months. Cytokine effects on EBV latency programs were defined by immunoblot for EBNA2, EBNA3C and LMP1, since this panel of EBV oncoproteins can be used to assign the latency program. Interestingly, most of these cytokines reduced EBNA2 and 3C expression, though results were the most pronounced for IL-21, which rapidly and robustly impaired EBNA2/3C expression (Fig 1B). By contrast, IL-10 and IL-21 upregulated LMP1 expression within 2 days of treatment (Fig 1B). Similar effects were observed in a second LCL, GM12881 (S1B Fig). IL-21 also suppressed EBNA2 and upregulated LMP1 in latency III Jijoye Burkitt cells (S1C Fig), suggesting generalizable effects on the latency III program. Consistent with prior reports, IL-21 did not hyper-induce LMP2A expression in either GM12878 or Kem III LCLs, indicating that IL-21 may fail to induce recruitment of an activator to the LMP2 promoter or to instead dismiss a repressor (S1D Fig).

We next performed RNA-seq analysis to systematically characterize IL-15 and IL-21 effects on EBV genome-wide expression, as representative of FDC vs Tfh cytokine signaling, respectively. After six days of treatment, IL-15 significantly decreased expression of multiple latency III genes, including EBNA2, EBNA3, EBNA-LP, LMP1 and LMP2s, but increased expression of a subset of lytic cycle genes, including immediate early BZLF1 and early BMRF1 (Fig 1C and S1 Table), suggestive of an abortive lytic cycle. Instead, IL-21 significantly increased abundance of LMP1 mRNA but decreased abundances of EBNA2, EBNA3, EBNA-LP, and LMP2 mRNAs (Fig 1D and S1 Table). Consistent with effects on EBNA2 and LMP1 expression, IL-15 downregulated the EBNA2 target gene CD300A, while IL-21 upregulated levels of the LMP1/NF-κB target ICAM-1 and downmodulated CD300A [21,39,40] (S1E and S1F Fig and S2 Table).

GC cytokines alter B-cell gene expression patterns via multiple effectors, including distinct JAK/STAT pathways. As expected, the panel of cytokines differentially activated STATs, including STAT5 activation by IL-2 and IL-15 versus STAT6 activation by IL-4 versus STAT3 activation by IL-6, IL-10, IL-21 and IL-27, as judged by immunoblot, using well characterized phosphorylation marks of STAT activation [26,27] (Figs 1E and S1B–S1C). Consistent with our RNA-seq analyses, IL-15 de-repressed BZLF1 and BMRF1 expression at the protein level, as did IL-2 (Fig 1E), suggesting that it induces an abortive lytic cycle in at least a subset of cells. Notably, these two cytokines share receptor beta and gamma chain subunits, which are transmembrane proteins that activate downstream pathways, including JAK/STAT [41].

EBV-infected B-cells likely also experience combinatorial cytokine signaling in germinal center microenvironments. To model these effects, we next treated GM12878 and GM12881 LCLs with the Tfh cytokines IL-4+IL-21, the FDC cytokine IL-15 together with IL-21 or the FDC cytokines IL-15 and IL-27 (Fig 1A). IL-15/IL-21 and to a lesser extent IL-15/IL-27 suppressed EBNA2 and EBNA3C expression somewhat more strongly than either cytokine alone. Interestingly, while IL-4/IL-21 did not repress either EBNA2 or EBNA3C more than IL-21 alone, IL-4/IL-21 did boost LMP1 levels beyond those observed with IL-21 or IL-4 alone (S2A and S2B Fig), likely due to alterations in JAK/STAT signaling.

We next asked the extent to which Tfh and FDC cues can alter EBV latency gene expression within the latency I B-cell context. While several Tfh signals, including IL-4+CD40L, IL-10 or IL-21 can each de-repress LMP1 expression in B-cells with the latency I program [30–34], it has remained unknown the extent to which other GC microenvironmental cues more broadly alter EBV latency gene expression within latency I. To gain insights, we treated latency I Mutu I and Kem I Burkitt cells with a panel of Tfh and FDC cytokines, as there is no primary human B-cell latency I models currently available. IL-21 strongly activated STAT3, as judged by tyrosine 705 phosphorylation, and robustly de-repressed LMP1 expression in Mutu I and Kem I (Figs 1F and S2C and S2D). By contrast, IL-4+CD40L or IL-10 treatment also induced STAT3 phosphorylation and LMP1 expression, albeit to a lesser extent (Fig 1F). This did not appear to be a full transition to the latency II program, as neither IL-10 nor IL-21 induced LMP2A to an appreciable degree in Mutu I or Kem I (S2E Fig). Differences between GC cytokine STAT activation in the latency I vs III context may relate to altered expression of receptors versus negative regulators of JAK/STAT signaling.

To then systematically analyze latency I B-cell responses to the Tfh signals IL-4+CD40L vs IL-21, we performed RNA-seq on Mutu I that were mock-stimulated or stimulated by these Tfh cues for 1 day. This early timepoint was chosen since we observed robust effects on LMP1 de-repression by that early timepoint, and as we observed reduced Mutu I viability with longer treatments. Consistent with our immunoblot analysis, IL-4+CD40L only modestly increased LMP1 expression, whereas IL-21 strongly induced LMP1 (Fig 1G and 1H). Notably, these stimuli did not significantly de-repress expression of EBNA or mildly increased LMP2 mRNAs, suggesting a specific effect at the level of the LMP1 promoter.

Analysis of Mutu I host transcriptome responses to either IL-4+CD40L or IL-21 treatment highlighted upregulation of multiple LMP1 target genes [42], including mRNAs encoding the NF-κB subunits RelB and p100/52 (encoded by NFKB2), ICAM-1 and IRF4 (S2F and S2G Fig). The NF-κB pathway signaling pathway and EBV infection were amongst the pathways most highly enriched by either cytokine treatment. While direct effects of the cytokines themselves may account for a subset of these changes, we note that IL-21 is not a strong inducer of NF-κB signaling, suggesting that de-repressed LMP1 may be an important mediator of the observed host transcriptomic changes.

### STAT3 and STAT5 mediate GC cytokine effects on the EBV latency III program

**Fig 2:**
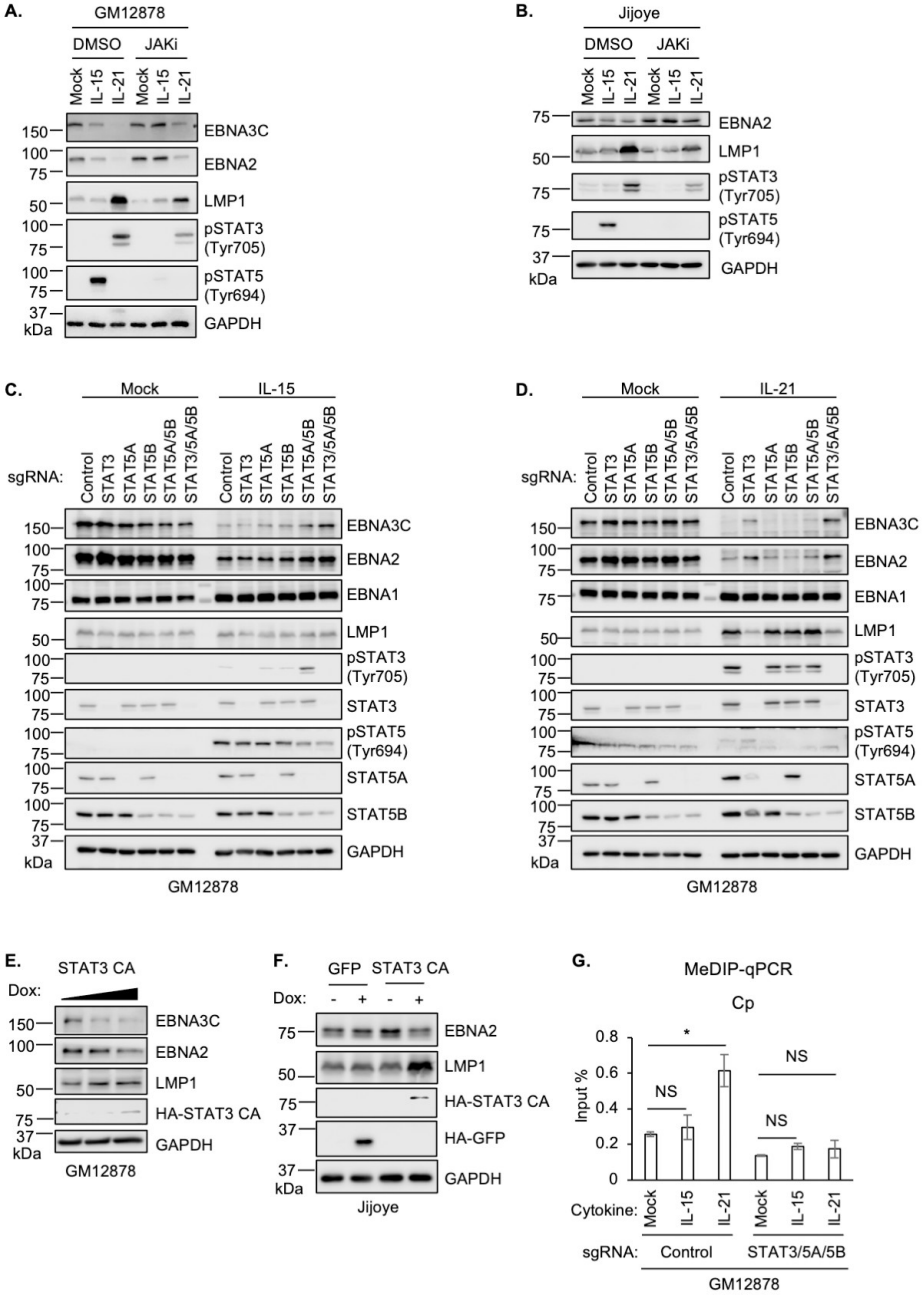
STAT3/5 roles in EBV latency gene regulation by IL-15 or IL-21. (**A-B**) Immunoblot analysis of WCL from GM12878 (A) or Jijoye (B) cells pre-treated with DMSO vehicle or JAK inhibitor CAS 457081-03-7 (JAKi, 200 ng/ml) for one hour, followed by treatment with IL-15 or IL-21 for six days. (**C-D**) Immunoblot analysis of WCL from GM12878 cells expressing control sgRNA versus sgRNA targeting the indicated STAT3 and/or STAT5 genes, which were then mock treated or treated with IL-15 (C) or IL-21 (D) for six days. (**E-F**) Immunoblot analysis of WCL from GM12878 (E) or Jijoye (F) cells expressing cDNA encoding GFP or constitutive activated STAT3 (STAT-CA), whose expression was induced by 0.5 or 1 μg/ml doxycycline (Dox). (**G**) Methylated DNA immunoprecipitation and quantitative PCR (MeDIP-qPCR) analysis of GM12878 cells with control or STAT3/5A/5B sgRNA expression, which were mock treated or treated with IL-15 or with IL-21 for six days. Shown is mean ± standard deviation (SD) from n = 3 replicates of Cp qPCR signal. *p < 0.05; NS: not significant. IL-15 and IL-21 were used at 100 ng/ml throughout. Immunoblots are representative of n = 3 replicates.

We next investigated effects of chemical or CRISPR JAK/STAT blockade to gain further insights into specific STAT roles in control of EBV latency gene expression downstream of IL-15 and IL-21. First, to broadly characterize JAK/STAT roles in LMP and EBNA expression, we treated latency III GM12878 and Jijoye cells with IL-15 or IL-21, in the absence or presence of the pan-JAK ATP-competitive inhibitor CAS 457081-03-7 (also referred to as JAK inhibitor I or JAKi). On-target JAKi effects were confirmed by immunoblot analysis of STAT3 and STAT5 phosphorylation, which demonstrated loss of STAT5 Tyrosine 694 and downmodulation of STAT3 Tyrosine 705 phosphorylation in IL-15 and IL-21 treated cells, respectively (Fig 2A and 2B). JAKi impaired IL-15 downmodulation of EBNA2 and 3C expression (Fig 2A and 2B). Likewise, JAKi treatment partially impaired IL-21 suppression of EBNA2 and EBNA3C expression and reduced the extent to which IL-21 hyper-induced LMP1 (Fig 2A and 2B). Incomplete blockade of IL-21 driven STAT3 phosphorylation may explain the comparatively milder JAKi effects on IL-21 than on IL15 regulation of latency III expression.

To examine individual STAT transcription factor roles downstream of IL-15 or IL-21, we next used CRISPR/Cas9 editing. Since IL-15 and IL-21 most robustly induced STAT5 and STAT3 phosphorylation (Fig 1E), we tested effects of CRISPR depletion of STAT3, of STAT5A or STAT5B isoforms [43], or of combinations thereof, given their potentially redundant roles. IL-15 repression of EBNA2 or 3C was not significantly perturbed by depletion of STAT3, STAT5A or STAT5B alone. However, concurrent GM12878 and Jijoye STAT5A/5B depletion impaired repression of EBNA2 and 3C by IL-15 and to a lesser extent by IL-21 (Figs 2C, 2D and S3A). In addition, concurrent CRISPR depletion of STAT3, STAT5A and STAT5B more strongly impaired EBNA3C repression by IL-15 (Fig 2C), suggestive of a partially redundant STAT3 and 5 roles, likely at the EBV C promoter.

STAT3 depletion was sufficient to block IL-21 driven LMP1 hyper-induction and impaired IL-21 driven EBNA2/EBNA3C repression (Fig 2D). Nonetheless, combined STAT3/5A/5B editing more strongly impaired EBNA2 and EBNA3C repression by IL-21 (Fig 2D). Despite robust STAT1 activation by IL-21 and to a lesser extent by IL-10 (Fig 1E), CRISPR STAT1 depletion did not alter IL-21 or IL-10 effects on EBNA or LMP1 expression (S3B and S3C Fig). STAT3 KO impaired EBNA2/3C repression and LMP1 hyper-induction downstream of IL-10 (S3B and S3C Fig). Taken together, these results suggest that STAT3 and 5 have partially redundant roles in cytokine mediated EBNA2/3C repression, perhaps through the action of STAT3/5 heterodimers, whereas STAT3 is a major driver of IL-21 driven LMP1 hyper-induction, with relevance to latency III to II reprogramming in the GC microenvironment.

We next tested effects of expressing a STAT3 constitutively active allele (STAT3-CA), in which STAT3 C-terminal loop SH2 domain A662C and N664C point mutations [44,45] drive constitutive STAT3 homodimerization, DNA binding and transcription activation. STAT3-CA expression diminished EBNA2 and increased LMP1 abundance in GM12878 and in Jijoye B-cells (Fig 2E and 2F), further suggesting that STAT3 plays a critical but opposite role in EBNA2/3 vs LMP1 regulation. These observations are consistent with a model in which GC cytokine signaling culminates in assembly of STAT3/5-containing transcriptional repressor complexes at the EBV genomic C promoter, but instead triggers formation of a STAT3 homodimer containing activator complex at the LMP1 promoter.

Since IL-15 and IL-21 upregulated the host transcriptional repressor BCL6 (S1E and S1F Fig) which plays major roles in GC B-cell biology and is critical for GC formation, we tested BCL6 roles in cytokine driven EBV latency gene expression. However, BCL6 CRISPR KO did not appreciably alter IL-21 effects on EBNA2 or LMP1 abundance (S4A Fig). BCL6 KO also did not affect IL-21 effects on LCL plasma membrane CD300A or ICAM-1, which are targets of EBNA2 and LMP1, respectively (S4B Fig).

DNA methylation is critical for suppression of Cp driven EBNA expression in B-cells with latency I, and presumably also in latency II [21,46–49]. We therefore used methylation DNA immunoprecipitation (MeDIP) and qPCR to characterize IL-15 versus IL-21 effects on LCL Cp DNA methylation levels. Notably, IL-21, but not IL-15 significantly increased C, LMP1 and LMP2 promoter methylation levels, and STAT3/5A/5B depletion reversed this effect (Figs 2G and S4C). These results indicate that STAT3/5 promote cross-talk between IL-21, C and LMP promoter DNA methylation.

### STAT and DNA methylation role in latency I LMP1 de-repression by GC cytokines

**Fig 3:**
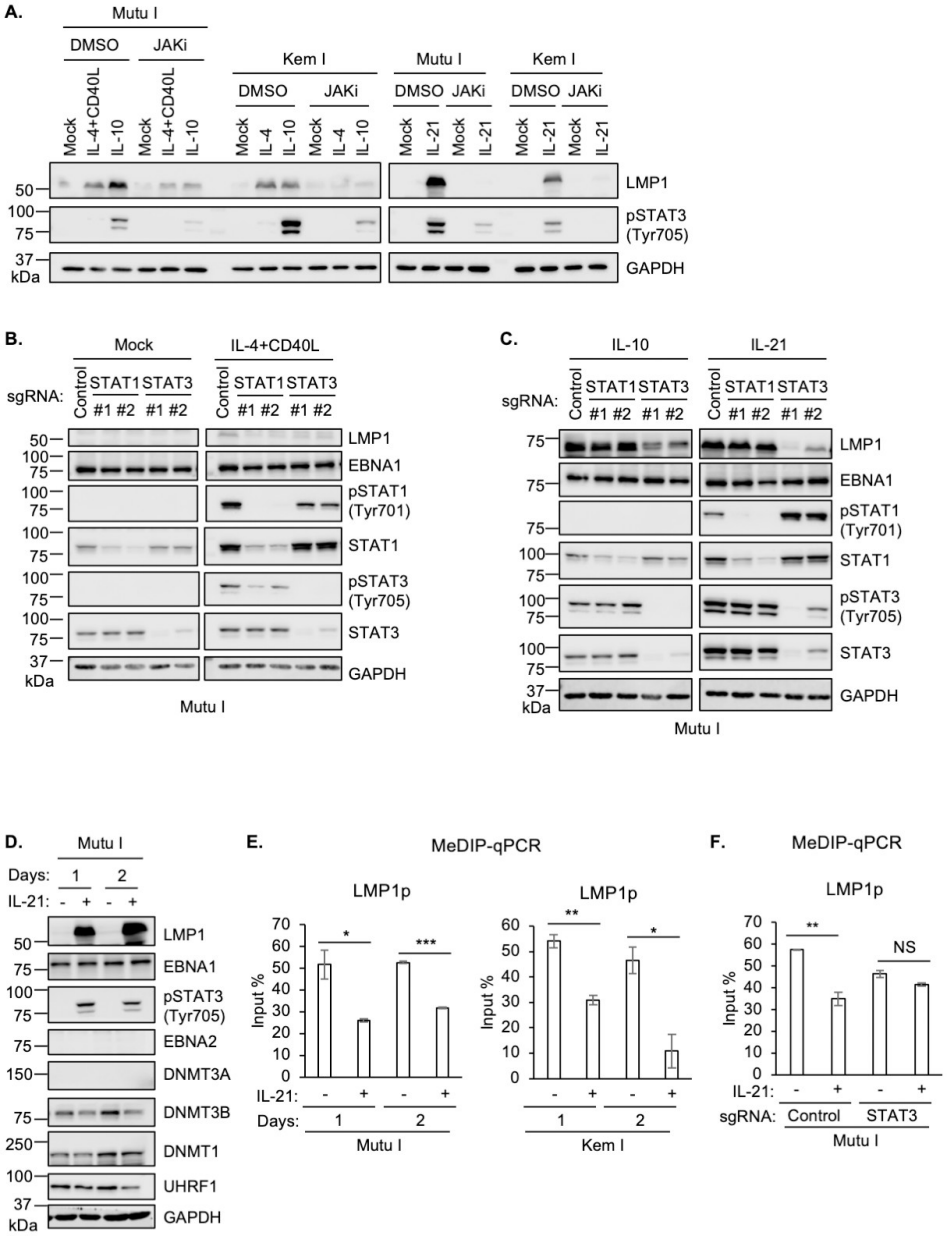
STAT3 roles in GC cytokine mediated LMP1 de-repression in latency I B-cells. (**A**) Immunoblot analysis of WCL from latency I Mutu I or Kem I B cells pre-treated with DMSO or JAKi (200 ng/ml) for one hour, followed by treatment with the indicated cytokines for one day. (**B**) Immunoblot analysis of WCL from Mutu I expressing control sgRNA or sgRNA targeting the indicated STAT transcription factor gene, mock treated or treated with IL-4+CD40L for one day. (**C**) Immunoblot analysis of WCL from Mutu I expressing control sgRNA or sgRNA targeting the indicated STAT transcription factor gene, treated with IL-10 or IL-21 for one day. (**D**) Immunoblot analysis of WCL from Mutu I mock treated or treated with IL-21 for one or two days. (**E**) MeDIP q-PCR analysis of LMP1 promoter methylation in Mutu I (left) or Kem I (right) mock treated or treated with IL-21 for one or two days. Shown are mean ± SD values of % input from n = 3 replicates. (**F**) MeDIP-qPCR analysis of LMP1p in Mutu I with control or *STAT3* targeting sgRNA, mock treated or IL-21 treated for one day. *p < 0.05; **p < 0.01; ***p < 0.001. Cytokines were used at 50 ng/ml. Blots are representative of n = 3 replicates.

To gain insights into JAK/STAT roles in GC cytokine triggered LMP1 de-repression in latency I B-cells, we treated Mutu I or Kem I Burkitt cells with IL-10, IL-21 or IL-4 together with CD40 ligand, in the absence or presence of JAK inhibition. We tested these GC stimuli since each hyper-induced LMP1 and robustly induced STAT phosphorylation in latency I cells and had previously been reported to de-repress LMP1 expression from latency I [30–36] (Fig 1F). JAKi treatment strongly impaired LMP1 upregulation by each of these stimuli (Figs 3A and S5A), consistent with a key JAK/STAT role in epigenetic regulation at the level of the LMP1 promoter.

To next gain mechanistic insights into specific STAT roles, we tested effects of CRISPR depletion of STAT transcription factors that were highly phosphorylated in response to these GC stimuli (Fig 1F). Depletion of either STAT1 or STAT3 blunted LMP1 de-repression by IL-4+CD40L stimulation or even by IL-4 alone (Figs 3B and S5B). By contrast, depletion of STAT3, but not STAT1, impaired IL-10 and IL-21 mediated LMP1 de-repression in Mutu I and Kem I cells (Figs 3C and S5B). Thus, STAT1/3 heterodimers may be important for IL-4 driven LMP1 de-repression, whereas distinct STAT3 heterodimers or homodimers may mediate LMP1 de-repression downstream of IL-10 and IL-21. Consistent with the latter hypothesis, induction of the constitutively active STAT3 allele was sufficient to de-repress LMP1 expression in Mutu I (S5C Fig). Likewise, STAT3 and to a somewhat lesser extent STAT6 over-expression enhanced LMP1 de-repression in response to cytokine treatment (S5D Fig).

To gain further insights into cytokine cross-talk with EBV-genomic CpG methylation, we next analyzed IL-21 effects on the abundance of DNA methyltransferase machinery. IL-21 downregulated expression of the *de novo* CpG methylation writer DNMT3B (Fig 3D), whose expression counteracts latency III gene expression [21]. Likewise, IL-21 downmodulated UHRF1 expression (Fig 3D), which is important for maintenance of EBV genomic methylation marks, together with DNMT1 [21]. Therefore, to further characterize IL-21 effects on CpG methylation of key EBV genomic promoters, we performed MeDIP-qPCR analysis on Mutu I or Kem I Burkitt cells treated with IL-21 for 1 or 2 days. Interestingly, IL-21 downmodulated the high level of DNA methylation at the LMP1 and C promoters, but not at the LMP2 promoter in either Mutu I or Kem I (Figs 3E and S5E). Additional epigenetic marks may maintain Cp and LMP2p silencing upon IL-21 stimulation in the latency I context, including those driven by STAT-containing repressive complexes. In support of a STAT3 role in modulation of LMP1p methylation downstream of IL-21, we did not observe diminished LMP1p methylation levels in STAT3 depleted Mutu I cells upon IL-21 treatment (Fig 3F).

### GC cytokine effects on LMP1 promoter histone epigenetic marks

**Fig 4:**
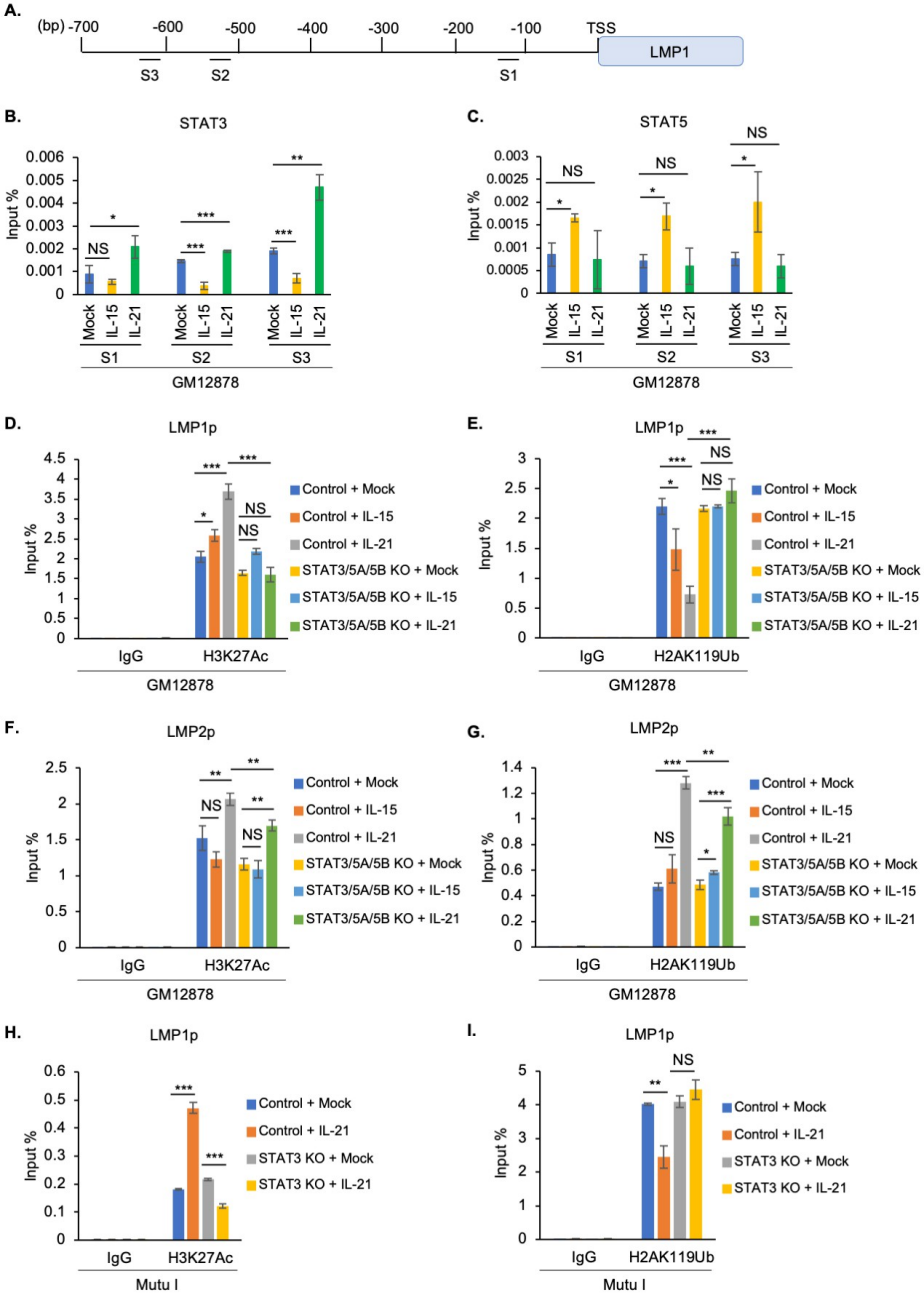
STAT 3 and 5 role in GC cytokine mediated latency III LMP1 promoter epigenetic remodeling. (**A**) Schematic diagram of LMP1 promoter STAT binding sites, S1, S2 and S3 [58]. (**B-C**) Chromatin immunoprecipitation (ChIP) qPCR analysis of STAT3 (B) or STAT5 (C) LMP1 promoter occupancy in GM12878 mock treated or treated with IL-15 or IL-21 for six days. (**D-E**) ChIP-qPCR analysis of LMP1 promoter H3K27Ac (D) or H2AK119Ub (E) epigenetic mark abundances in GM12878 expressing control versus STAT3/5A/5B targeting sgRNA, mock treated or treated with IL-15 or IL-21 for six days. (**F-G**) ChIP-qPCR analysis of LMP2 promoter H3K27Ac (F) or H2AK119Ub (G) abundances in GM12878 expressing control versus STAT3/5A/5B targeting sgRNA, mock treated or treated with IL-15 or IL-21 for six days. (**H-I**) ChIP-qPCR analysis of LMP1 promoter H3K27Ac (H) or H2AK119Ub (I) abundances in Mutu I expressing control sgRNA versus STAT3 targeting sgRNA, mock treated or treated with IL-21 for one day. All ChIP results are presented as % input mean ± SD from n = 3 replicates. *p < 0.05; **p < 0.01; ***p < 0.001.

In addition to DNA methylation, histone epigenetic marks strongly contribute to EBV latency gene expression [14,21,50–57]. We therefore next profiled GC cytokine effects on the LMP1 promoter. Since previous studies identified three LMP1 promoter sites occupied by STAT factors [58] (Fig 4A), we performed chromatin immunoprecipitation (ChIP) and qPCR analyses in LCLs mock treated or treated with FDC-derived IL-15 or Tfh-derived IL-21. IL-21 increased STAT3 occupancy at the S3 site, located at approximately 600 base pairs (bp) upstream of LMP1p, and to a lesser extent at the S2 and S1 sites, located at approximately 500 and 100 bp upstream of LMP1p (Fig 4B). Interestingly, IL-15 instead downmodulated STAT3 occupancy at S2 and S3, consistent with the observation that it does not hyper-induce LMP1 in latency III. By contrast, IL-15 but not IL-21 significantly increased STAT5 occupancy at S1-S3 (Fig 4C). These results further support the hypothesis that IL-21 driven STAT3, and potentially STAT3 homodimers, are major drivers of LMP1 hyper-induction.

To characterize STAT roles in LMP1 promoter histone epigenetic regulation, we next performed ChIP-qPCR analysis in control versus CRISPR-edited LCLs. In control LCLs, IL-15 and to a greater extent IL-21 increased LMP1p histone 3 lysine 27 acetylation (H3K27Ac), a mark which correlates with promoter activation. By contrast, IL-15 and IL-21 failed to upregulate LMPp H3K27Ac level in LCLs depleted for STAT3, STAT5A and STAT5B (Fig 4D). Since we recently found a role for the polycomb repressive complex (PRC1) I histone 2A lysine 119 ubiquitin (H2AK119Ub) mark in repression of LMP1 expression [21], we next examined GC cytokine and STAT roles on LMP1p H2AK119Ub levels in LCLs. IL-15 and to a greater extent IL-21 significantly diminished H2AK119Ub abundance in control, but not STAT3/5A/5B KO GM12878 (Fig 4E). Despite lack of appreciable LMP2A hyper-induction, IL-21 nonetheless increased H3K27Ac levels in GM12878 control and STAT3/5A/5B edited LCLs (Fig 4F). Interestingly, IL-21 hyper-induced H2AK119Ub repressive marks at LMP2p in both control and STAT3/5A/5B edited cells (Fig 4G). Given PRC1 roles in repression of LMP expression, this result suggests a potential mechanism by which LMP1 but not LMP2A is hyper-induced in IL-21 treated B-cells, and are consistent with a model in which STAT3/5 occupy LMP1 but not LMP2 promoter sites.

We did not observe decreases in the repressive histone 3 lysine lysine 9 dimethyl (H3K9me2) or trimethyl (H3K9me3) marks with either IL-15 or IL-21 treatment in control or STAT KO LCLs (S6A and S6B Fig). However, repressive histone 3 lysine 27 trimethyl (H3K27me3) repressive marks increased somewhat upon IL-15 or IL-21 treatment in STAT3/5A/5B triple edited LCLs (S6C Fig). Similar effects were observed at the LMP2 promoter, though IL-21 increased the repressive H3K9me3 mark in both control and STAT3/5A/5B edited cells (S6D–SF Fig), potentially contributing to the lack of IL-21 driven LMP2A hyper-induction.

We next characterized IL-21 epigenetic effects on the LMP1 promoter in latency I B-cells, given the observation that IL-21 strongly activates STAT3 phosphorylation and de-represses LMP1 expression, whereas other GC cytokine stimuli did so comparatively weakly. As anticipated, IL-21 significantly increased H3K27Ac at the LMP1 promoter in Mutu I cells (Fig 4H). Interestingly, STAT3 was necessary for this IL-21 driven epigenetic remodeling, as STAT3 depletion prevented IL-21 driven H3K27Ac activating mark at the LMP1 promoter (Fig 4H). Similarly, IL-21 significantly diminished the repressive H2AK119Ub and H3K9me2 marks at the LMP1 promoter in a STAT3 dependent manner (Figs 4I and S7A). By contrast, IL-21 did not significantly alter repressive H3K9me3 or H3K27me3 marks at the latency I LMP1 promoter (S7B Fig). Interestingly, IL-21 did not significantly alter activating or repressive histone marks at the Mutu I LMP2 promoter (S7C–S7F Fig). These results indicate that the absence of STAT3 signaling is important for silencing LMP1 expression in latency I, with relevance to the transition from latency II to latency I.

### GC cytokines remodel epigenetic status of C promoter

Multiple GC cytokines repressed latency III EBNA expression, suggestive of epigenetic effects at the level of Cp, which drives the large EBV transcript encoding all six EBNAs.

**Fig 5:**
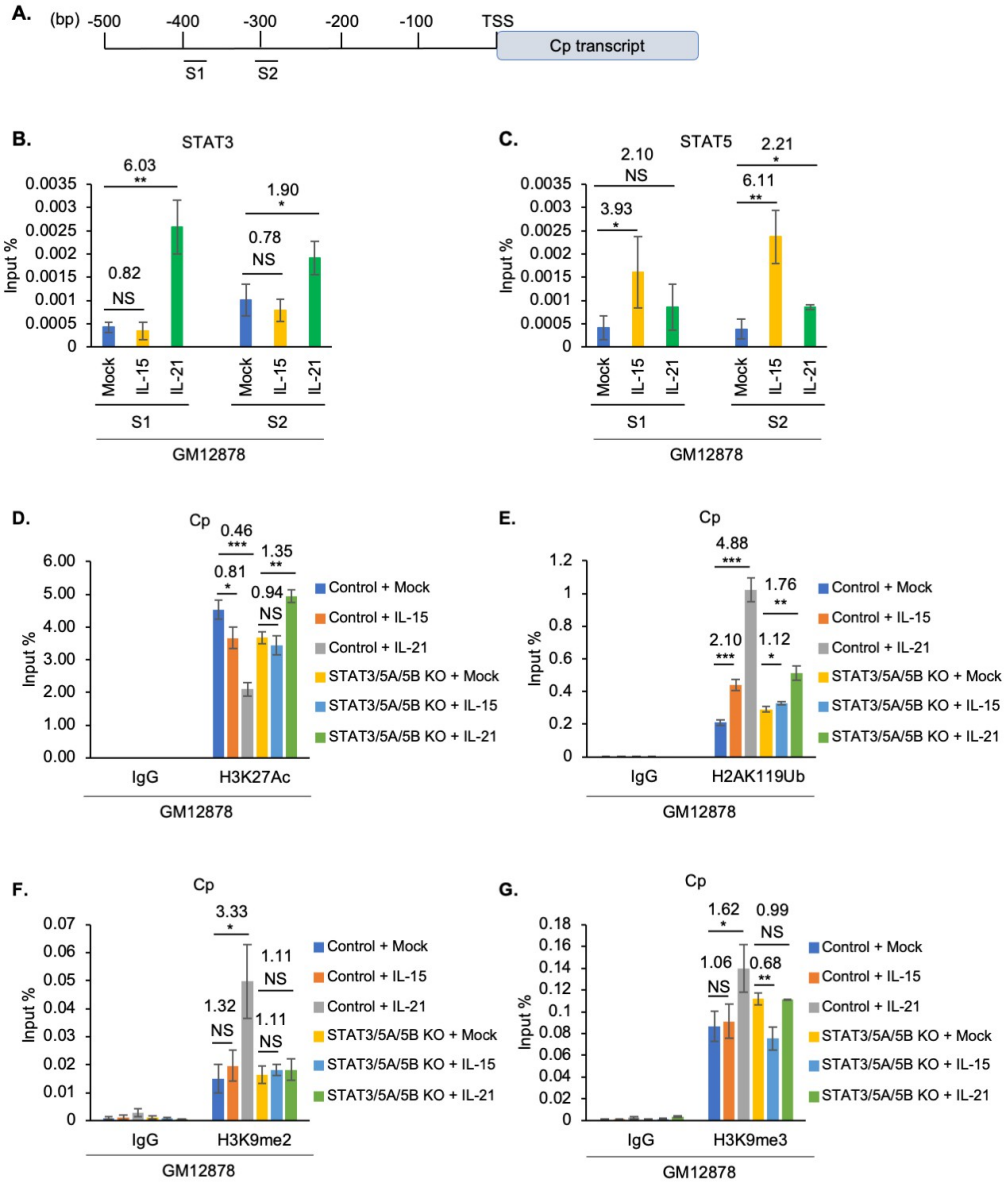
STAT3 and 5 role in GC cytokine mediated C promoter epigenetic remodeling. (**A**) Schematic diagram of PROMO [59,60] predicted STAT binding sites on C promoter. (**B-C**) ChIP-qPCR analysis of STAT3 (B) or STAT5 (C) C promoter occupancy in GM12878 mock treated or treated with IL-15 or IL-21 for six days. (**D-G**) ChIP-qPCR analysis of Cp H3K27Ac (D), H2AK119Ub (E), H3K9me2 (F) and H3K9me3 (G) abundances in GM12878 expressing control versus STAT3/5A/5B targeting sgRNA, mock treated or treated with IL-15 or IL-21 for six days. All ChIP results are presented as % input mean ± SD from n = 3 replicates. Relative fold changes to Mock treated cells are labeled. *p < 0.05; **p < 0.01; ***p < 0.001.

We performed ChIP to characterize how IL-15 and IL-21 alter STAT3 versus STAT5 occupancy at two predicted STAT binding sites using PROMO online tool [59,60], located at 300 and 400 bp upstream of Cp (Fig 5A). Consistent with our observation that IL-15 and IL-21 predominantly activated STAT5 versus STAT3 in latency III cells, respectively (Figs 1B and S1B and S1C), IL-21 but not IL-15 significantly upregulated STAT3 occupancy at both the S1 and S2 sites upstream of Cp (Fig 5B). Conversely, IL-15 significantly induced STAT5 occupancy at both S1 and S2, whereas IL-21 weakly induced STAT5 binding to S2 (Fig 5C). Taken together with our CRISPR and immunoblot analyses, these data are compatible with a model in which a STAT5 or STAT3 homodimer and to a lesser extent a STAT3/5 heterodimer are critical for IL-15 or IL-21 mediated Cp repression.

At the epigenetic level, ChIP-qPCR assays highlighted that IL-21 more strongly reduced H3K27Ac marks at Cp than IL-15 (Fig 5D). Consistent with key STAT3 and 5 roles in GC cytokine driven epigenetic remodeling at Cp, CRISPR editing of STAT3/5A/5B blocked H3K27Ac loss at Cp in GM12878 stimulated by either IL-15 or IL-21 (Fig 5D). Similarly, IL-15 and to a greater extent IL-21 increased H2AK119Ub repressive marks at Cp, and CRISPR STAT3/5A/5B editing blunted cytokine-driven H2AK119Ub deposition (Fig 5E). Likewise, IL-21 but not IL-15 significantly increased deposition of the H3K9me2 and H3K9me3 repressive marks at Cp, and this increase was blunted by STAT3/5 editing (Fig 5F and 5G). Interestingly, neither IL-15 nor IL-21 increased repressive H3K27me3 marks at Cp, arguing against PRC2 roles in their repression of Cp (S8A Fig). By comparison, Cp is silenced in latency I, and likely related to that, we observed relatively small differences in the Cp epigenetic status in control or STAT3 edited Mutu I at rest or following IL-21 treatment (S8B–S8F Fig). These results are consistent with a model in which STAT3 nucleate transcription co-activator complexes at LMP1p but STAT3 and 5 mediates repressive complexes at Cp in latency III B-cells, and that latency I cells maintain the ability to respond to STAT-dependent epigenetic remodeling at LMP1p.

### JAK/STAT signaling roles in newly infected B-cell latency gene expression and transformation

**Fig 6:**
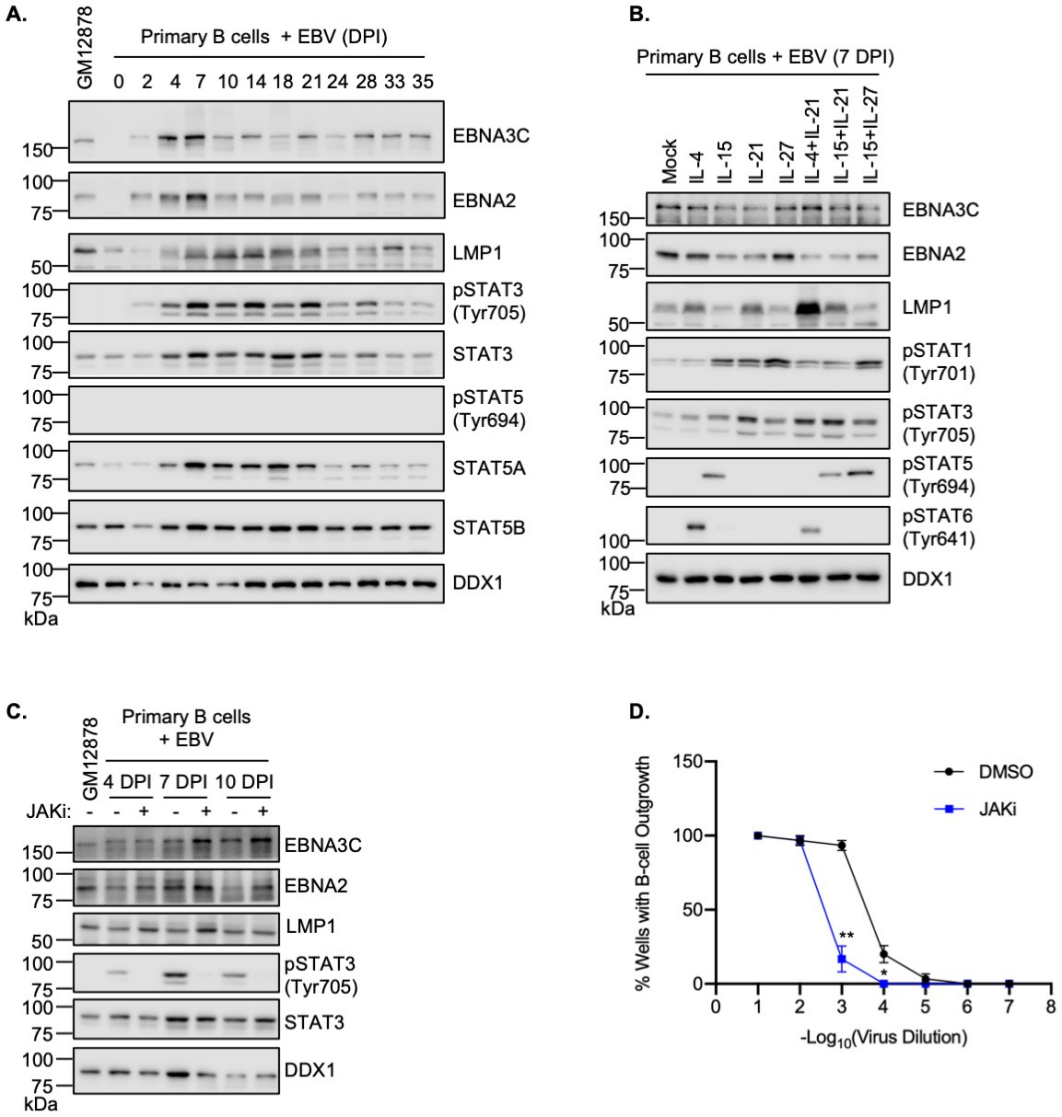
IL-15 and IL-21 remodeling of latency III gene expression in newly infected primary human B cells. (**A**) Immunoblot analysis of WCL from primary human B cells at the indicated days post infection (DPI) by the Akata EBV strain. (**B**) Immunoblot analysis of WCL from primary human B cells at 7 DPI, which were then mock treated or treated with the indicated cytokines for six days. (**C**) Immunoblot analysis of WCL from primary B cells that were treated with DMSO or JAKi (200 ng/ml) for two days at 4, 7 or 10 DPI. GM12878 WCL was included as a control. (**D**) Primary human B-cell transformation assay characterizing effects of DMSO vs JAKi (200 ng/ml) treatment on primary human B-cell outgrowth following infection by Akata EBV. Fitted non-linear regression curves are presented as mean ± SD from n = 3 replicates, *p < 0.05; **p < 0.01. Blots are representative of n = 3 replicates. Cytokines were used at 100 ng/ml.

JAK/STAT signaling contributes to EBV latency gene expression in newly infected primary human B-cells [61,62]. Notably, STAT3 tyrosine 705 phosphorylation is elevated within 30 minutes of primary B-cell exposure to EBV, and total STAT3 protein levels are upregulated within 8 hours of EBV infection [62,63]. Such early effects on STAT3 are likely triggered by EBV interaction with B-cell receptors and/or internalization and preceded EBV latency gene expression [62,63]. To build upon these data, we characterized STAT3 and STAT5 phosphorylation levels over multiple subsequent timepoints, in which EBV converts primary human cells into lymphoblastoid cells. Purified CD19+ human peripheral blood B cells were isolated by negative selection and infected with EBV. Timecourse analysis was then performed to measure levels of EBV latency gene, STAT3 and STAT5 expression, as well as of STAT3 and 5 phosphorylation, to indicate their activation status. Interestingly, EBV upregulated STAT3 and STAT5A levels, in particular between days 4 and 21 post-infection, whereas STAT5B levels were relatively constant. Whereas EBV triggered STAT3 phosphorylation, in particular between days 4 and 21 post-infection, a period in which LMP1 levels were markedly elevated and EBNA2 and 3C levels diminished (Fig 6A). Notably, EBV did not trigger STAT5 phosphorylation, as judged by immunoblot of phosphotyrosine 694 (Fig 6A).

We next investigated the effects of IL-21 on EBV latency gene expression when dosed at day 7 post-infection, the earliest timepoint when B-cells begin to convert to lymphoblastoid physiology [9,64]. IL-21 reduced EBNA2 and 3C expression and hyper-induced LMP1 (S9A Fig), suggesting conserved STAT roles in EBV latency gene expression in newly infected cells and in LCLs. We next tested the effects of IL-15 and IL-21 on EBV latency gene expression at day 10 post-infection. Treatment with either cytokine for 6 days reduces EBNA2 expression (S9B Fig). IL-21 treatment also strongly down-modulated EBNA2 target gene CD23 [65,66] abundance when applied at multiple distinct timepoints between days 2 and 35 post-infection (S9C and S9D Fig).

To then gain insights into germinal center cytokine effects on EBV-infected primary B-cells, we treated cells at 7 days post-infection with vehicle control, IL-4, IL-15, IL-21, IL-27 or multiple combinations thereof. IL-15 or IL-21 were each sufficient to reduce EBNA2 and EBNA3C expression relative to levels in untreated cells, but we did not appreciate additive effects of combinatorial cytokine treatment on their expression (Fig 6B). While IL-4 did not appreciably alter EBNA2 or EBNA3C levels, it was able to boost LMP1 protein expression above control cell levels. Interestingly, the combination of Tfh cytokines IL-4 and IL-21 hyper-induced LMP1 expression, whereas addition of the FDC cytokine IL-15 to IL-21 did not appreciably alter LMP1 levels relative to those observed with IL-21 treatment alone. Combinatorial treatment with the FDC cytokines IL-15 and IL-27 did not boost LMP1 levels relative to untreated cells (Fig 6B). Immunoblot analysis demonstrated the expected phosphorylation of STAT1, 3, 5 and 6 in response to each of these cytokines, suggesting that their pathways were similarly activated by cytokines in the primary cell as well as transformed B cell settings (Fig 6B).

**Fig 7:**
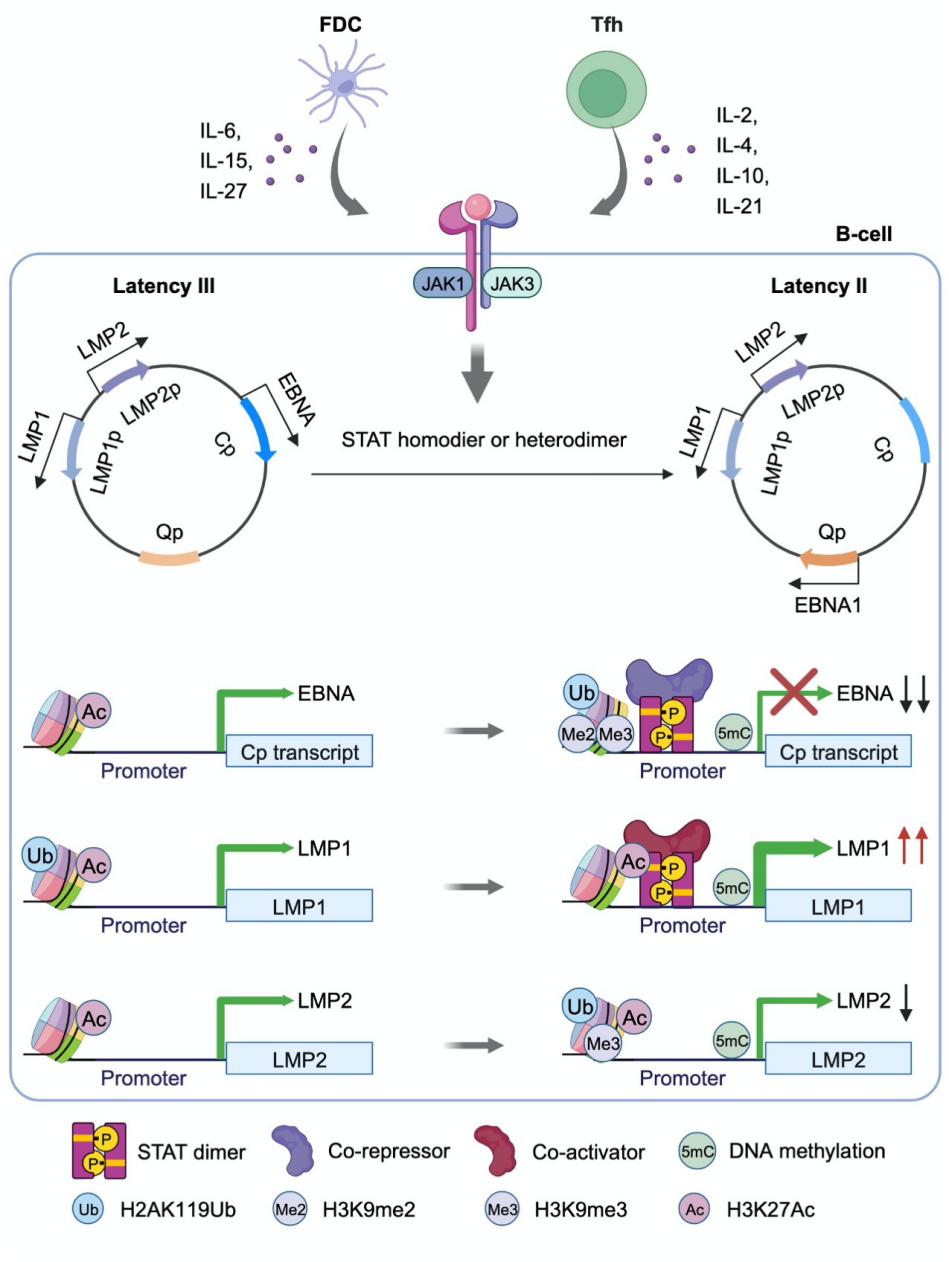
Model of EBV latency promoter epigenetic remodeling by GC cytokine driven JAK/STAT signaling. Cytokines secreted by germinal center FDC and Tfh cells trigger JAK/STAT signaling to drive STAT homodimer or heterodimer nuclear translocation. STAT dimers bind to the C promoter together with a co-repressor to increase levels of repressive 5-methylcytosine (5-mC), H3K9me2/3 and H2AK119Ub epigenetic marks, while decreasing activating H3K27Ac marks. C promoter inactivation decreases latency III EBNA expression. By contrast, STAT dimers bind to the LMP1 promoter together with a co-activator to drive epigenetic remodeling to increase levels of LMP1 promoter H3K27Ac and to decrease levels of H2AK119Ub, which supports LMP1 expression despite concurrently increased 5mC levels. By contrast, GC cytokines increase the abundances of LMP2 promoter repressive H2AK119Ub, H3K9me3 and 5mC marks to downmodulate LMP2 expression from levels observed in latency III. (BioRender was used to create the schematic models).

To characterize the roles of JAK/STAT signaling in EBV-mediated B-cell transformation, we treated newly infected primary human B-cells with JAKi at 4, 7 or 10 DPI. Consistent with JAK/STAT downmodulation of EBNA2 and EBNA3 expression at these early times post-infection, JAKi treatment increased EBNA2 and EBNA3C expression (Fig 6C). Surprisingly, JAKi treatment also mildly increased LMP1 expression, which likely occurred secondary to increases in EBNA2 levels. JAKi treatment also impaired outgrowth of EBV-infected B-cells in a transformation assay (Fig 6D), suggesting that EBV-driven JAK/STAT signaling supports B-cell immortalization, potentially by titrating the levels of EBV oncoprotein expression. Taken together, our results support a model in which JAK/STAT signaling exerts control over EBV latency gene expression through epigenetic effects on key EBV latency gene promoters (Fig 7).

## Discussion

The EBV germinal center model posits that microenvironmental cues trigger latency program remodeling in order to support infected B-cell survival, immunoevasion and memory B-cell differentiation [6]. Yet, knowledge has remained incomplete about how specific Tfh and FDC signals alter EBV latency gene promoter epigenomes. Here, we present the first CRISPR analyses to dissect specific STAT roles in latently EBV-infected B-cell responses to GC cytokine cues. We highlight crosstalk between EBV genomic STAT occupancy, histone modification and DNA methylation in GC-cytokine driven reprograming. Our data support a model in which GC cytokines drive a STAT3/5 dependent transcription repressive complex at the EBV genomic C promoter, but instead drive a STAT3 dependent transcription activation complex at the LMP1 promoter (Fig 7). STAT3/5 heterodimers may serve to nucleate a transcription repressive complex at Cp, whereas STAT3 homodimers may instead promote transcription activation at LMP1p. Since STAT3 homodimers support EBNA1 expression in latency II [58,67], STAT signaling provides a key means by which EBV-infected cells translate GC microenvironmental cues to the epigenome.

We recently identified that DNA methylation is sufficient for silencing Cp-driven EBNA expression, but that DNA methylation and PRC1 are each important for silencing LMP1 and LMP2A expression in latency I Burkitt cells [8,21]. It is therefore noteworthy that IL-21 increased C, LMP1 and LMP2 promoter DNA methylation, but decreased the PRC1 H2AK119Ub mark only at the LMP1 promoter in a STAT3/5 dependent manner. IL-21 may alter LMP1 promoter H2AK119Ub abundance by promoting dismissal of PRC1 from LMP1p or instead by recruiting the H2AK119Ub erasers BAP1 or USP16 [68] in a STAT3/5 dependent manner. Importantly, EBNA2 induces and also recruits the TET2 demethylase to the C and LMP promoters [69,70]. Therefore, EBNA2 downmodulation by IL-21 likely contributed to the observed increase in EBV genomic methylation.

How then is LMP2A supported in the GC microenvironment upon GC cytokine driven EBNA2 repression? While we studied responses to individual cytokine cues, it is possible that combinatorial signals may be needed to support LMP2A expression. Alternatively, a distinct GC microenvironmental cue not modelled in our study may be required to support LMP2A expression, such as from dendritic or regulatory T cells. Thus, a prediction of this model is that on a single cell level, subsets of EBV-infected cells may express LMP1, LMP1 together with LMP2A, or perhaps only LMP2A within distinct GC microenvironmental niches. Such flexibility may support evasion from cytotoxic T-cell responses directed at either LMP1 or LMP2A, may alter the extent of infected cell proliferation or residence time within the GC, and/or may support GC exit upon memory B-cell differentiation into the EBV memory cell reservoir.

Latent EBV infection supports B-cell JAK/STAT signaling, which may provide a basal level to calibrate latency gene expression, even in the absence of Tfh or FDC derived cytokines. The LMP1 C-terminal activation region 3 binds to JAK3 [71], though this region of LMP1 may not by itself be sufficient to activate JAK/STAT signaling [72,73]. LMP1 induces IL-10 expression *in vitro* [74] and together with LMP2A in germinal center B-cells *in vivo* [75], though levels are likely to be lower than those secreted by Tfh in the GC microenvironment. Furthermore, EBV driven reactive oxygen species accumulation further supports STAT3 activation in the early stages of EBV-driven B-cell outgrowth [61]. Thus, EBV may have evolved to require a high threshold of JAK/STAT signaling to ensure that latency program selection occurs in the GC microenvironment on the pathway to memory cell differentiation.

While our data suggest that STAT3 is critical for GC cytokine induced remodeling towards latency II, it is noteworthy that IL-21 more strongly induced LMP1 than IL-6, IL-10 or IL-27, which also strongly activated STAT3. One model to reconcile these observations is that IL-21 signaling may induce a higher abundance of STAT3 homodimers within latency III cells, and these are required for the observed effects on LMP1 expression. Alternatively, IL-21 may more strongly induce co-activators that together with STAT3 upregulate LMP1 expression.

In addition to its roles in EBV latency gene regulation, STAT3 represses lytic gene expression of multiple human herpesviruses, including herpes simplex virus 1 (HSV-1), EBV, and Kaposi’s sarcoma-associated herpesvirus (KSHV) [62,76–79]. For instance, STAT3 supports the expression of KAP1/TRIM28 and the deposition of repressive histone 3 lysine 9 trimethyl (H3K9me3) marks to repress EBV and KSHV lytic reactivation [78,79]. Therefore, it will be of interest to examine whether STAT3 recruits KAP1 to repress C promoter. Moreover, STAT3 also plays major roles in EBV-driven oncogenic B-cell growth. For instance, B-cells from patients with STAT3 hypomorphic mutation resist EBV-mediated immortalization [62,63]. Likewise, transgenic B cell LMP1 expression accelerates lymphomagenesis in a murine model, in which tumors exhibited elevated STAT3 activity [80]. Elevated STAT3 signaling was also observed in mice with transgenic LMP1 and LMP2A B-cell co-expression [81]. Relatedly, activated JAK/STAT signaling is observed in EBV+ diffuse large B-cell lymphoma [82], the Hodgkin lymphoma Reed-Sternberg tumor cell [58], post-transplant lymphoproliferative disease [83–85] and plasmablastic lymphoma [86].

Gamma-herpesviruses may have evolved to subvert STAT3 signaling to support GC-dependent differentiation. EBV, Kaposi’s Sarcoma Associated Herpesvirus and murine gammaherpesvirus 68 (MHV68) have each evolved mechanisms to activate STAT3 [62,79,87–91]. STAT3 is important for the establishment of longterm latency by MHV68 [92]. However, in contrast to our findings for EBV, STAT3 does not directly regulate MHV68 viral gene expression, but instead dampens type I IFN responses in newly infected B-cells [93]. Thus, EBV has evolved specific mechanisms to coopt B-cell STAT signaling to modulate latency gene expression in response to B-cell cues. It is not presently known whether EBV+ B-cells enter GC dark zone structures, in which B-cells undergo multiple rounds of proliferation and somatic hypermutation following stimulation by Tfh and FDC within light zone regions. Since GC cytokine stimulation and STAT3 phosphorylation take place within light zones [94], it is plausible that EBV+ B-cells may express higher LMP1 levels within light zones, and that they may therefore predominantly reside within GC light zone regions. However, single cell analyses of EBV-infected secondary lymphoid tissue have not yet been performed to address this open area.

Many EBV-associated cancers are currently treated with high intensity chemotherapy or radiation therapy, which causes substantial morbidity and increases the risk of secondary malignancies. Likewise, use of anti-CD20 B-cell depleting antibodies such as rituximab to target EBV-driven lymphoproliferative diseases can be complicated by lymphopenia, variable B-cell recovery, hypogammaglobulinemia, prolonged neutropenia and rare but fatal viral reactivation [95,96]. Therefore, novel therapeutic approaches are needed to more selectively target a range of EBV-associated cancers. In depth understanding of the molecular mechanisms that control EBV latency gene expression may lay the foundation for rational therapeutic approaches that target specific EBV-associated cancers, either by depriving tumor cells of necessary EBV oncogene expression in the case of lymphomas with the latency III program or by de-repressing immunogenic latency protein antigens in tumors with more highly restricted forms of latency, such as Burkitt lymphoma.

With regards to tumors dependent on latency III such as post-transplant lymphoproliferative disease or central nervous system lymphoma, it may be feasible to target JAK/STAT signaling to downmodulate EBNA expression. An intriguing possibility is that targeting the JAK/STAT pathway with clinically approved small-molecule inhibitors such as ruxolitinib, currently used treatment of graft-versus-host disease [97] and for other hematological disorders, could be tested as a prophylactic or preemptive approach for the prevention of EBV-driven PTLD. While EBV-driven lymphomas can emerge in patients treated with potent T-cell immunosuppression together with ruxolitinib, it might nonetheless confer protection against the outgrowth of EBV-driven polymorphic B-cell clones.

Conversely, cytokine therapy or epigenetic approaches could be developed to derepress LMP1 in order to sensitize latency I tumors such as Burkitt lymphoma to antiviral T-cell surveillance, including adoptive transfer of T-cells reactive with LMP1 derived epitopes [98,99]. Not only is LMP1 itself a targetable tumor antigen, but once de-repressed, it also stimulates potent T-cell responses to tumor associated antigens [100]. In addition, IL-15 and IL-21 enhance natural killer and CD8+ T cell cytotoxicity and antitumor effects [101–103]. An interesting approach could therefore be to combine HLA-matched, third party cytotoxic CD8+ T-cells [104] with IL-21, perhaps as an Fc-fusion to enhance [105]. Indeed, IL-21 therapy appears to generally be well tolerated [106]. In this manner, IL-21 treatment could not only induce LMP1 expression in Burkitt tumors, but could also enhance cellular immune responses to EBV-infected tumor cells.

Bispecific antibodies that target the T-cell receptor (TCR) and an HLA-presented LMP2A peptide is a promising approach which has demonstrated activity against latency III EBV+ B-cell murine xenografts [107]. It may therefore be possible to combine IL-21 with a bispecific antibody targeting the TCR and LMP1 derived peptides to target EBV+ Burkitt tumors. Further studies are required to explore whether IL-21 or other germinal center cytokines might likewise de-repress or boost LMP1 expression in EBV+ epithelial cell tumors, including gastric and nasopharyngeal carcinoma.

IL-27 receptor signaling was recently found to play a key role in the stimulation of CD8+ T-cell anti-EBV responses, as congenital biallelic loss-of-function variants in the IL-27 receptor alpha chain are associated with severe primary EBV infection. Interestingly however, EBV-transformed B-cells secrete IL-27, and an autocrine loop between IL-27 receptor+ T-cells and IL-27 secretion by infected B-cells is important for EBV-transformed B-cell proliferation *in vitro* [108]. Thus, the IL-27 receptor/IL-27 axis could potentially constitute another therapeutic target. Further studies will be required to determine whether there may be a role for IL-27 therapy, for instance to boost anti-EBV T-cell responses. While IL-27 supports EBV-transformed B-cell proliferation at doses secreted by infected cells in culture, we found that at high doses, IL-27 instead downmodulates EBNA expression, which might also provide anti-tumor effects by impairing transformed cell growth *in vivo*.

In summary, multiple FDC and Tfh derived cytokines repress Cp driven EBNA expression, whereas IL-21 and to a lesser extent IL-4 and IL-10 support LMP1 expression through STAT dependent EBV epigenomic remodeling. STAT3 and 5 were critical for cytokine mediated Cp silencing, whereas STAT3 was critical for LMP1 hyper-induction. GC cytokine signaling increased repressive epigenetic marks, including DNA methylation and H2AK119Ub, while decreased active chromatin mark H3K27Ac at Cp in EBV latency III cells. However, IL-21 increased H3K27Ac at the LMP promoters, but decreased H2AK119Ub only at the LMP1 promoter in both EBV latency III and I cells. IL-21 also decreased DNA methylation at LMP1 promoter in EBV latency I Burkitt cells. Therefore, STAT3 and 5 serve as major hubs of EBV epigenomic remodeling in response to GC cytokine signaling to support latency program remodeling.

## Materials and methods

### Ethics statement

Primary B-cells were isolated from discarded, de-identified peripheral blood mononuclear cells from the Brigham and Women’s Hospital Blood Bank, obtained following platelet donation, using an Institutional Review Board approved protocol and written consent was obtained from the donors.

### Cell culture

EBV+ latency I cells, P3HR1 (A gift from Dr. Elliott Kieff), Akata (A gift from Dr. Elliott Kieff), Mutu I (A gift from Dr. Jeffrey Sample) and Kem I (A gift from Dr. Jeffrey Sample), and latency III cells, GM12878 (purchased from Coriell Institute), GM12881 (purchased from Coriell Institute), Kem III (A gift from Dr. Jeffrey Sample) and Jijoye (purchased from American Type Culture Collection, ATCC), were all grown in Roswell Park Memorial Institute (RPMI) 1640 medium with 10% fetal bovine serum (FBS). 293T cells (purchased from ATCC) were grown in Dulbecco’s Modified Eagle’s Medium (DMEM) with 10% FBS. All B cell lines used in this study stably express *Streptococcus pyogenes* Cas9, which were generated by lentiviral transduction followed by blasticidin selection [109]. All cells were grown in a humidified chamber with 5% carbon dioxide at 37°C.

### Cytokines and JAKi treatment

Latency I and III B cells were seeded at 500,000 cells/ml in 12-well plates and mock treated with PBS or cytokines (S3 Table) at 50 ng/ml and 100 ng/ml, respectively. EBV infected human primary B cells were treated with IL-15 or IL-21 at 100 ng/ml. For long-term treatment, cells were re-seeded with fresh culture medium supplemented with indicated cytokines, which were refreshed every 48 hours. For JAK inhibitor treatment, cells were pre-treated with JAK inhibitor I (JAKi) at indicated doses for one hour at 37 °C, followed by cytokine treatment, refreshed every 48 hours.

### CRISPR/Cas9 editing

CRISPR/Cas9 editing was performed as previously described [110,111]. In brief, Brunello library [112] single guide RNA (sgRNA) were cloned into pLentiGuide-puro (a gift from Feng Zhang, Addgene plasmid #52963), pLenti-spBsmBI-sgRNA-Hygro (a gift from Rene Maehr, Addgene plasmid #62205), or pLentiGuide-zeo (a gift from Rizwan Haq, Addgene plasmid #160091). sgRNA sequences were verified by Sanger sequencing. All sgRNAs used in this study are listed in S4 Table. Target cells were transduced with lentivirus expressing sgRNAs against the target gene, or as a control, against GFP (pXPR-011, a gift from John Doench). Lentivirus were produced by transfection of 293T cells with pCMV-VSV-G (a gift from Bob Weinberg, Addgene plasmid #8454), psPAX2 (a gift from Didier Trono, Addgene plasmid #12260), and the sgRNA expression vector using the TransIT-LT1 transfection reagent. 293T supernatants were added to target B-cells at 48 and 72 hours post-293T transfection. Transduced cells were then selected with puromycin (3 μg/ml) for 3 days.

For STAT5A and STAT5B combinatorial editing, GM12878 or Jijoye Cas9+ cells were initially transduced with lentivirus expressing STAT5A sgRNA and selected with hygromycin (200 μg/ml) for 7 days, followed by transduction with lentiviruses expressing STAT5B sgRNA and selected with puromycin (3 μg/ml) for 3 days. For experiments with STAT5A/5B/3 editing, STAT3 was depleted in STAT5A/5B edited GM12878 cells by transduction with lentivirus expressing STAT3 sgRNA, and transduced cells were selected by zeomycin (200 μg/ml) for 7 days. On-target CRISPR effects were validated by immunoblotting.

### cDNA cloning and transduction

cDNA entry vectors used in this study are listed in S4 Table, which were purchased from DNASU and Addgene. STAT1, 3, and 6 cDNA were sub-cloned into the destination vector pLX-TRC313 (a gift from John Doench) and STAT3_p.A662C_N664C (constitutively active STAT3 with A662C and N664C mutations) [44] was cloned into pLIX-402 (a gift from John Doench) by Gateway LR recombination. As described previously [113], the destination vector and donor vector containing the gene of interest were co-incubated with 1x LR Clonase Enzyme Mix overnight at room temperature. The reaction mixture was then transformed into Stbl3 competent cells and plated on LB agar plate containing ampicillin. Destination vectors were used to make lentiviruses, which were used to transduce target B-cells. Transduced cells were selected by puromycin or hygromycin for pLIX-402 or pLX-TRC313 vectors, respectively.

### Immunoblotting

Immunoblotting analysis was performed as previously described [21], Cells were lysed in 1x Laemmli Sample Buffer and sonicated briefly. For detection of LMP2A, cells were lysed with M-PER Mammalian Protein Extraction Reagent and incubated on ice for 30 minutes. Lysates were centrifuged at 15,000 x g for 15 minutes. 2x Laemmli Sample Buffer was added into supernatant and boiled at 70 °C for 10 minutes. Lysates were resolved by SDS-PAGE and transferred onto nitrocellulose membranes, which were blocked with 5% nonfat milk in TBST buffer for 1 hour and then incubated with primary antibodies at 4 °C overnight. Blots were then washed 3 times with TBST, followed by secondary antibody incubation for 1 hour at room temperature. Blots were washed 3 times in TBST buffer and were developed with the ECL chemiluminescence substrate. Images were captured by a LI-COR Fc platform. All antibodies used in this study are listed in S3 Table.

### Flow cytometry assay

Cells were washed once with FACS buffer (2% FBS v/v, PBS), followed by incubation with primary antibodies in FACS buffer for 30 minutes at room temperature in the dark. Labeled cells were palleted, washed twice and resuspended in FACS buffer into flow cytometry-compatible tubes and processed immediately. Flow cytometry data was recorded with a BD FACSCalibur instrument and analyzed with FlowJo X software.

### Akata virus production, primary B cells isolation and infection

EBV was produced from EBV+ Akata cells. In brief, EBV+ Akata cells were resuspended in FBS-free RPMI media at 2–3 million cells/mL and induced with 0.25% (v/v) goat anti-human immunoglobulin G serum for 6 hours at 37 °C. Cells were then pelleted and resuspended in 4% FBS RPMI media and cultured in 37 °C for 3 days. Supernatant were then collected and filtered through 0.45 μM filter. Viruses were 50-fold concentrated by ultracentrifugation and stored at -80 °C until use.

Primary B-cells were isolated by negative selection from discarded, de-identified peripheral blood mononuclear cells from the Brigham and Women’s Hospital Blood Bank, obtained following platelet donation, using an Institutional Review Board approved protocol and written consent was obtained from the donors. RosetteSep and EasySep negative isolation kits were used according to the manufacturer’s instructions to isolate CD19+ B-cells. B cells were then cultured with RPMI containing 10% FBS. Primary B cells were seeded at 500,000 cells/ml and infected by the Akata EBV strain at multiplicity of infection (MOI) of 0.1, as determined by the Green Daudi assay.

### Primary human B cell EBV transformation assay

EBV transformation assays were performed as described previously [114]. Briefly, purified human primary B cells were infected with Akata EBV using serial 10-fold dilutions. Cells were cultured with media containing DMSO or JAKi (200 ng/ml) and were seeded in 96-wells plates at 500,000 cells/ml (30 wells per condition). Media containing DMSO or JAKi was refreshed every three to four days. The percentage of wells positive for B-cell outgrowth at four weeks post infection was calculated and plotted relative to the dilution of virus.

### Chromatin immunoprecipitation (ChIP) assay

After cytokine treatment, 10 million cells were cross-linked with 1% formaldehyde in 10 ml growth medium for 10 minutes, followed by quenching with 2.5M glycine in distilled water for 5 minutes. Cells were washed with ice-cold PBS three times and then lysed in 0.5 ml 1% SDS lysis buffer (50 mM Tris, 10 mM EDTA, 1% SDS), supplemented with 1x cOmplete, EDTA-free Protease Inhibitor Cocktail. Chromatin was fragmented using a Bioruptor Pico sonication device with 30s on/ 30s off (20 cycles for GM12878 cells, 12 cycles for Mutu I cells), and centrifuged at 13,200 rpm for 10 mins at 4 °C. This protocol resulted in fragments of average length 100–200 bp, to enable differentiation of STAT occupancy at closely spaced EBV genomic STAT binding sites. Supernatants were removed and then diluted 1:10 in ChIP dilution buffer (1.2 mM EDTA, 16.7 mM Tris, 167 mM NaCl, 0.01% SDS, 1.1% Triton X-100) supplemented with protease inhibitor cocktail. Chromatin from one million cells was used for each ChIP reaction. 1% of sonicated chromatin was saved as input and stored at -80 °C until use. Diluted chromatin was rotated overnight at 4 °C with the indicated antibody and 20 μl protein A+G magnetic beads. Next day, beads were pelleted, washed twice with a lower salt buffer (150 mM NaCl, 2 mM EDTA, 20 mM Tris, 0.1% SDS, 1% Triton X-100) and then a high-salt buffer (500 mM NaCl, 2 mM EDTA, 20 mM Tris, 0.1% SDS, 1% Triton X-100), and once with LiCl buffer (0.25 M LiCl, 1% NP-40, 1% sodium deoxycholate, 1 mM EDTA, 10 mM Tris) and finally TE buffer (10 mM Tris, 1 mM EDTA). Chromatin was eluted in Elution buffer (100 mM NaHCO3, 1% SDS) and reverse cross-linked at 65 °C for 2 hours. QIAquick PCR purification kits were used to purify the immunoprecipitated DNA, followed by qPCR with PowerUp SYBR green PCR master mix on a CFX Connect Real-Time PCR Detection System (Bio-Rad). All reagents, antibodies and primers used for ChIP are listed in S3 and S4 Tables.

### Methylated DNA immunoprecipitation (MeDIP) assay

Genomic DNA was extracted using DNeasy Blood& Tissue Kit, followed with MeDIP assay with MagMeDIP kit, following the manufacturer’s protocol. qPCR was then performed with primers specifically target EBV promoters. All reagents and primers used for MeDIP are listed in S3 and S4 Tables.

### RNA-seq and data analysis

mRNA was isolated via the RNeasy Mini kit with in-column genomic DNA digestion protocol was followed, according to the manufacturer’s instructions. To construct indexed libraries, 1 μg of total RNA was used for polyA mRNA purification, using the NEBNext Poly(A) mRNA Magnetic Isolation Module, followed by library preparation using the NEBNext Ultra RNA Library Prep with Sample Purification Beads. Each experimental treatment was performed in biological triplicate. Libraries were multi-indexed, pooled and sequenced on an Illumina NovaSeq 6000 using PE150 Sequencing Strategy by Novogene Corporation. Adaptor-trimmed reads were mapped to Akata EBV genome (Accession#: KC207813.1) or human GRCh37.83 transcriptome assembly using salmon (v1.10.0). Quality control was performed using fastqc. Differentially expressed genes were identified in R (v4.0.3) using DESeq2 [115] under default settings with the apeglm shrinkage estimator (https://doi.org/10.1093/bioinformatics/bty895) and annotations derived from the hg19 build from Ensembl release 75 [116] and accessed via biomaRt. Volcano plots were generated in GraphPad Prism 8, using Log_2_ (Fold Change) and −Log_10_ (p value) data. Differentially expressed genes from each condition were subjected to Enrichr analysis and top 10 KEGG pathways with adjusted *p* value < 0.05 cutoff were visualized. All RNA-seq datasets were deposited to the NIH GEO omnibus with accession number GSE252480. All reagents and kits used for RNA-seq are listed in S3 Table.

### Quantification and statistical analysis

All immunoblots were performed with three independent experiments and qPCR was performed in three independent experiments. Statistical significance was assessed with Student’s t test using GraphPad Prism 8 software, where NS = not significant, *p* > 0.05; * *p* < 0.05; ** *p* < 0.01; *** *p* < 0.001. Biorender was used to create the schematic models.

## Supporting information

S1 Fig GC cytokine effects on latency III B-cell EBV and host gene expression. (**A**) Schematic of Tfh and FDC cytokine driven JAK/STAT signaling. (BioRender was used to create the schematic models.) (**B-C**) Immunoblot analysis of WCL from GM12881 (B) and latency III Jijoye (C) cells treated with the indicated cytokines for six days. (**D**) Immunoblot analysis of WCL from GM12878 and Kem III cells six days post mock, IL-15 or IL-21 treatment. (**E**) Volcano plot (left) and KEGG pathway analysis (right) of host genes expression in GM12878 stimulated by IL-15 versus mock-simulated for six days from n = 3 independent replicates. The top 10 most differentially expressed KEGG pathways are shown. (**F**) Volcano plot (left) and KEGG pathway analysis (right) of host genes expression in GM12878 stimulated by IL-21 versus mock-simulated for six days from n = 3 independent replicates. Cytokines were used at 100 ng/ml and were refreshed every two days. Immunoblots are representative of n = 3 replicates.
(TIFF)

S2 Fig GC cytokine effects on latency III and I B-cell EBV and host gene expression. (**A**-**B**) Immunoblot analysis of WCL from GM12878 (A) or GM12881 (B) LCLs that were mock treated or treated with the indicated cytokines for six days. Densitometry values of GAPDH normalized EBNA3C, EBNA2 and LMP1 levels are shown beneath each row. (**C**) Immunoblot analysis of WCL from latency I Kem I Burkitt B cells treated with the indicated cytokines for 24 hours. (**D**) Immunoblot analysis of WCL from Mutu I and Kem I treated with IL-21 for one or two days, as indicated. (**E**) Immunoblot analysis of WCL from Mutu I and Kem I one day post mock, IL-10 or IL-21 treatment. GM12878 WCL was included as a positive control. (**F**) Volcano plot (left) and KEGG pathway analysis (right) of differentially expressed Mutu I host genes one day after IL-4+CD40L vs mock stimulation from n = 3 independent replicates. (**G**) Volcano plot (left) and KEGG pathway analysis (right) of differentially expressed Mutu I host genes one day after IL-4+CD40L vs mock stimulation from n = 3 independent replicates. The top 10 KEGG pathways amongst differentially regulated genes are shown. Cytokines and CD40L were used at 100 ng/ml and 50 ng/ml for EBV latency III and I cells, respectively. Immunoblots are representative of n = 3 replicates.
(TIFF)

S3 Fig STAT3 and 5 roles in IL-15 and IL-21 driven EBV latency III gene regulation. (**A**) Immunoblot analysis of WCL from latency III Jijoye B cells expressing control sgRNA or sgRNA targeting STAT5A and STAT5B, mock treated or treated with IL-15 or IL-21 for six days. (**B-C**) Immunoblot analysis of WCL from Jijoye (B) or GM12878 (C) expressing control sgRNA or sgRNA targeting the indicated STAT transcription factor gene, mock treated or treated with IL-10 or IL-21 for six days. Blots are representative of n = 3 replicates. Cytokines were used at 100 ng/ml and refreshed every 2 days.
(TIFF)

S4 Fig IL-21 effects on LCL EBNA2 and LMP1 expression are not dependent on BCL6 but correlate with STAT-dependent LMP promoter methylation. (**A**) Immunoblot analysis of WCL from GM12878 cells expressing control sgRNA or independent *BCL6* targeting sgRNA that were mock treated or treated with IL-21 (100 ng/ml) for two or four days. Blot is representative of n = 3 replicates. (**B**) Flow cytometry analysis of LMP1 target ICAM-1 and EBNA2 target CD300A plasma membrane expression in GM12878 expressing control sgRNA or BCL6 sgRNA and mock treated or IL-21 treated for 2 or 4 days, as indicated. (**C**) MeDIP-qPCR analysis of GM12878 expressing control or sgRNA targeting STAT3/5A/5B, mock treated or treated with IL-15 or IL-21 for six days, followed by qPCR with primers targeting the LMP1 promoter (LMP1p, left) or LMP2 promoter (LMP2p, right). Mean ± SD ChIP-qPCR % input values from n = 3 replicates are shown. *p < 0.05; **p < 0.01; ***p < 0.001.
(TIFF)

S5 Fig STAT roles in LMP1 de-repression in GC cytokine treated latency I B cells. (**A**) Immunoblot analysis of WCL from Mutu I cells treated with JAKi (0–1,000 ng/ml) for one hour, followed by IL-21 treatment for one or two days. GM12878 cell lysate was included as a positive control. (**B**) Immunoblot analysis of WCL from Kem I expressing control sgRNA or sgRNA targeting STAT1 or STAT3, mock treated or treated with the indicated cytokine for one day. (**C**) Immunoblot analysis of WCL from Mutu I conditionally induced for control GFP or constitutively active STAT3 for one day by 0.5 or 1 μg/ml doxycycline. (**D**) Immunoblot analysis of Mutu I expressing the indicated control GFP or STAT cDNA and stimulated as indicated for 1 day. (**E**) MeDIP-qPCR of the LMP2 promoter (left) and C promoter (right) in Mutu I and Kem I, mock treated or IL-21 treated for one day. Mean ± SD input % of n = 3 replicates are shown, *p < 0.05; **p < 0.01. All cytokines were used at 50 ng/ml. Blots are representative of n = 3 replicates.
(TIFF)

S6 Fig STAT roles in IL-15 and IL-21 driven LMP1 and LMP2 promoter epigenetic remodeling. (**A-C**) ChIP-qPCR analysis of LMP1 promoter H3K9me2 (A), H3K9me3 (B) or H3K27me3 (C) abundances from GM12878 expressing control or STAT3/5A/5B targeting sgRNAs, mock treated or treated with 100ng/ml IL-15 or IL-21 for six days. (**D-F**) ChIP-qPCR analysis of LMP2 promoter H3K9me2 (D), H3K9me3 (E) or H3K27me3 (F) abundances in GM12878 expressing control or STAT3/5A/5B targeting sgRNAs, mock treated or treated with IL-15 or IL-21 for six days. Mean ± SD input % of n = 3 replicates are shown, *p < 0.05; **p < 0.01.
(TIFF)

S7 Fig STAT3 roles in LMP1 and LMP2 promoter IL-21 driven epigenetic remodeling in latency I B-cells. (**A-B**) ChIP-qPCR analysis of LMP1 promoter H3K9me2 (A) or H3K9me3 and H3K27me3 (B) abundances from Mutu I expressing control or STAT3 targeting sgRNA, mock treated or treated with IL-21. (**C-F**) ChIP-qPCR analysis of LMP2 promoter H3K27Ac (C) or H2AK119Ub (D), H3K9me2 (E) or H3K9me3 and H3K27me3 (F) abundances in Mutu I expressing control or STAT3 targeting sgRNAs, mock treated or treated with IL-21. Cells were treated with 50 ng/ml IL-21 for one day. Mean ± SD input % of n = 3 replicates are shown, *p < 0.05; **p < 0.01.
(TIFF)

S8 Fig STAT3 roles in IL-15 and IL-21 driven Cp epigenetic remodeling. (**A**) ChIP-qPCR analysis of Cp H3K27me3 abundances in Mutu I expressing control or STAT3/5A/5B targeting sgRNAs, mock treated or treated with IL-15 or IL-21 (100ng/ml) for six days. (**B-F**) ChIP-qPCR analysis of Cp H3K27Ac (B), H2AK119Ub (C), H3K9me2 (D), H3K9me3 (E) or H3K27me3 (F) abundances in Mutu I expressing control or STAT3 targeting sgRNAs, mock treated or treated with IL-21 50 ng/ml for 1 day. Mean ± SD input % of n = 3 replicates are shown, *p < 0.05; **p < 0.01.
(TIFF)

S9 Fig IL-21 effects on newly EBV infected primary B-cell EBNA2 target gene CD23 expression. (**A**) Immunoblot analysis of WCL from primary human B cells at 7 DPI, which were then mock treated or stimulated with IL-21 for six days. (**B**) Immunoblot analysis of WCL from primary B cells at 10 DPI, mock treated or treated with IL-15 or IL-21 for six days. (**C**) Plasma membrane CD23 abundances in primary human B-cell mock treated or treated with IL-21 (100 ng/ml) at Day 7 vs 18 post-infection by Akata EBV. IL-21 was refreshed every 2 days. (**D**) Mean ± SD CD23 abundances from n = 3 replicates of primary B-cells infected by Akata EBV in the absence or presence of IL-21, as in (C), ***p < 0.001. Blots are representative of n = 3 replicates.
(TIFF)

S1 Table RNA-seq of EBV Gene Expression. (XLSX)

S2 Table RNA-seq of Host Gene Expression. (XLSX)

S3 Table Reagents, Antibodies and Kits. (XLSX)

S4 Table sgRNAs, plasmids and primers. (XLSX)

## References

[1] Farrell PJ. Epstein-Barr Virus and Cancer. Annu Rev Pathol. 2019;14:29–53. doi: 10.1146/annurev-pathmechdis-012418-013023 30125149

[2] Young LS, Yap LF, Murray PG. Epstein-Barr virus: more than 50 years old and still providing surprises. Nat Rev Cancer. 2016;16(12):789–802. doi: 10.1038/nrc.2016.92 27687982

[3] Gewurz B, Longnecker R, Cohen J. Epstein-barr virus. Fields Virology. 2021;2:7.

[4] Bjornevik K, Cortese M, Healy BC, Kuhle J, Mina MJ, Leng Y, et al. Longitudinal analysis reveals high prevalence of Epstein-Barr virus associated with multiple sclerosis. Science. 2022;375(6578):296–301. doi: 10.1126/science.abj8222 35025605

[5] Lanz TV, Brewer RC, Ho PP, Moon JS, Jude KM, Fernandez D, et al. Clonally expanded B cells in multiple sclerosis bind EBV EBNA1 and GlialCAM. Nature. 2022;603(7900):321–7. doi: 10.1038/s41586-022-04432-7 35073561 PMC9382663

[6] Thorley-Lawson DA. EBV Persistence—Introducing the Virus. Curr Top Microbiol Immunol. 2015;390(Pt 1):151–209. doi: 10.1007/978-3-319-22822-8_8 26424647 PMC5125397

[7] Buschle A, Hammerschmidt W. Epigenetic lifestyle of Epstein-Barr virus. Semin Immunopathol. 2020;42(2):131–42. doi: 10.1007/s00281-020-00792-2 32232535 PMC7174264

[8] Guo R, Gewurz BE. Epigenetic control of the Epstein-Barr lifecycle. Curr Opin Virol. 2022;52:78–88. doi: 10.1016/j.coviro.2021.11.013 34891084 PMC9112224

[9] Price AM, Luftig MA. To be or not IIb: a multi-step process for Epstein-Barr virus latency establishment and consequences for B cell tumorigenesis. PLoS Pathog. 2015;11(3):e1004656. doi: 10.1371/journal.ppat.1004656 25790223 PMC4366242

[10] Minarovits J. Human tumor viruses: induction of three-dimensional alterations in the host genome structure. Front Microbiol. 2023;14:1280210. doi: 10.3389/fmicb.2023.1280210 37928671 PMC10620758

[11] Chakravorty A, Sugden B, Johannsen EC. An Epigenetic Journey: Epstein-Barr Virus Transcribes Chromatinized and Subsequently Unchromatinized Templates during Its Lytic Cycle. J Virol. 2019;93(8). doi: 10.1128/JVI.02247-18 30700606 PMC6450099

[12] Kempkes B, Ling PD. EBNA2 and Its Coactivator EBNA-LP. Curr Top Microbiol Immunol. 2015;391:35–59. doi: 10.1007/978-3-319-22834-1_2 26428371

[13] Pich D, Mrozek-Gorska P, Bouvet M, Sugimoto A, Akidil E, Grundhoff A, et al. First Days in the Life of Naive Human B Lymphocytes Infected with Epstein-Barr Virus. mBio. 2019;10(5). 10.1128/mBio.01723-19 PMC6751056 31530670

[14] Zhou H, Schmidt SC, Jiang S, Willox B, Bernhardt K, Liang J, et al. Epstein-Barr virus oncoprotein super-enhancers control B cell growth. Cell Host Microbe. 2015;17(2):205–16. doi: 10.1016/j.chom.2014.12.013 25639793 PMC4539236

[15] Szymula A, Palermo RD, Bayoumy A, Groves IJ, Ba Abdullah M, Holder B, et al. Epstein-Barr virus nuclear antigen EBNA-LP is essential for transforming naive B cells, and facilitates recruitment of transcription factors to the viral genome. PLoS Pathog. 2018;14(2):e1006890. 29462212 10.1371/journal.ppat.1006890 PMC5834210

[16] Hong T, Parameswaran S, Donmez OA, Miller D, Forney C, Lape M, et al. Epstein-Barr virus nuclear antigen 2 extensively rewires the human chromatin landscape at autoimmune risk loci. Genome Res. 2021;31(12):2185–98. doi: 10.1101/gr.264705.120 34799401 PMC8647835

[17] Zhao B, Zou J, Wang H, Johannsen E, Peng CW, Quackenbush J, et al. Epstein-Barr virus exploits intrinsic B-lymphocyte transcription programs to achieve immortal cell growth. Proc Natl Acad Sci U S A. 2011;108(36):14902–7. doi: 10.1073/pnas.1108892108 21746931 PMC3169132

[18] Schlee M, Krug T, Gires O, Zeidler R, Hammerschmidt W, Mailhammer R, et al. Identification of Epstein-Barr virus (EBV) nuclear antigen 2 (EBNA2) target proteins by proteome analysis: activation of EBNA2 in conditionally immortalized B cells reflects early events after infection of primary B cells by EBV. J Virol. 2004;78(8):3941–52. doi: 10.1128/jvi.78.8.3941-3952.2004 15047810 PMC374249

[19] Kaiser C, Laux G, Eick D, Jochner N, Bornkamm GW, Kempkes B. The proto-oncogene c-myc is a direct target gene of Epstein-Barr virus nuclear antigen 2. J Virol. 1999;73(5):4481–4. doi: 10.1128/JVI.73.5.4481-4484.1999 10196351 PMC104340

[20] Saha A, Robertson ES. Mechanisms of B-Cell Oncogenesis Induced by Epstein-Barr Virus. J Virol. 2019;93(13). doi: 10.1128/JVI.00238-19 30971472 PMC6580952

[21] Guo R, Zhang Y, Teng M, Jiang C, Schineller M, Zhao B, et al. DNA methylation enzymes and PRC1 restrict B-cell Epstein-Barr virus oncoprotein expression. Nat Microbiol. 2020;5(8):1051–63. doi: 10.1038/s41564-020-0724-y 32424339 PMC7462085

[22] MacLennan IC. Germinal centers. Annu Rev Immunol. 1994;12:117–39. doi: 10.1146/annurev.iy.12.040194.001001 8011279

[23] Jandl C, King C. Cytokines in the Germinal Center Niche. Antibodies (Basel). 2016;5(1). 10.3390/antib5010005 PMC6698856 31557986

[24] Tangye SG, Ma CS. Regulation of the germinal center and humoral immunity by interleukin-21. J Exp Med. 2020;217(1). doi: 10.1084/jem.20191638 31821441 PMC7037251

[25] Quast I, Dvorscek AR, Pattaroni C, Steiner TM, McKenzie CI, Pitt C, et al. Interleukin-21, acting beyond the immunological synapse, independently controls T follicular helper and germinal center B cells. Immunity. 2022. doi: 10.1016/j.immuni.2022.06.020 35896116

[26] Rochman Y, Spolski R, Leonard WJ. New insights into the regulation of T cells by gamma(c) family cytokines. Nat Rev Immunol. 2009;9(7):480–90. doi: 10.1038/nri2580 19543225 PMC2814538

[27] Morris R, Kershaw NJ, Babon JJ. The molecular details of cytokine signaling via the JAK/STAT pathway. Protein Sci. 2018;27(12):1984–2009. doi: 10.1002/pro.3519 30267440 PMC6237706

[28] Hu X, Li J, Fu M, Zhao X, Wang W. The JAK/STAT signaling pathway: from bench to clinic. Signal Transduct Target Ther. 2021;6(1):402. doi: 10.1038/s41392-021-00791-1 34824210 PMC8617206

[29] Hu Q, Bian Q, Rong D, Wang L, Song J, Huang HS, et al. JAK/STAT pathway: Extracellular signals, diseases, immunity, and therapeutic regimens. Front Bioeng Biotechnol. 2023;11:1110765. doi: 10.3389/fbioe.2023.1110765 36911202 PMC9995824

[30] Kis LL, Salamon D, Persson EK, Nagy N, Scheeren FA, Spits H, et al. IL-21 imposes a type II EBV gene expression on type III and type I B cells by the repression of C- and activation of LMP-1-promoter. Proc Natl Acad Sci U S A. 2010;107(2):872–7. doi: 10.1073/pnas.0912920107 20080768 PMC2818931

[31] Konforte D, Simard N, Paige CJ. Interleukin-21 regulates expression of key Epstein-Barr virus oncoproteins, EBNA2 and LMP1, in infected human B cells. Virology. 2008;374(1):100–13. doi: 10.1016/j.virol.2007.12.027 18222514

[32] Kis LL, Nishikawa J, Takahara M, Nagy N, Matskova L, Takada K, et al. In vitro EBV-infected subline of KMH2, derived from Hodgkin lymphoma, expresses only EBNA-1, while CD40 ligand and IL-4 induce LMP-1 but not EBNA-2. Int J Cancer. 2005;113(6):937–45. doi: 10.1002/ijc.20654 15514968

[33] Kis LL, Takahara M, Nagy N, Klein G, Klein E. IL-10 can induce the expression of EBV-encoded latent membrane protein-1 (LMP-1) in the absence of EBNA-2 in B lymphocytes and in Burkitt lymphoma- and NK lymphoma-derived cell lines. Blood. 2006;107(7):2928–35. doi: 10.1182/blood-2005-06-2569 16332968

[34] Kis LL, Gerasimcik N, Salamon D, Persson EK, Nagy N, Klein G, et al. STAT6 signaling pathway activated by the cytokines IL-4 and IL-13 induces expression of the Epstein-Barr virus-encoded protein LMP-1 in absence of EBNA-2: implications for the type II EBV latent gene expression in Hodgkin lymphoma. Blood. 2011;117(1):165–74. doi: 10.1182/blood-2010-01-265272 20876453

[35] Takahara M, Kis LL, Nagy N, Liu A, Harabuchi Y, Klein G, et al. Concomitant increase of LMP1 and CD25 (IL-2-receptor alpha) expression induced by IL-10 in the EBV-positive NK lines SNK6 and KAI3. Int J Cancer. 2006;119(12):2775–83. doi: 10.1002/ijc.22139 17013900

[36] Nagy N, Adori M, Rasul A, Heuts F, Salamon D, Ujvari D, et al. Soluble factors produced by activated CD4+ T cells modulate EBV latency. Proc Natl Acad Sci U S A. 2012;109(5):1512–7. doi: 10.1073/pnas.1120587109 22307606 PMC3277165

[37] Gosselin J, TomoIu A, Gallo RC, Flamand L. Interleukin-15 as an activator of natural killer cell-mediated antiviral response. Blood. 1999;94(12):4210–9. 10590066

[38] Sharif-Askari E, Fawaz LM, Tran P, Ahmad A, Menezes J. Interleukin 15-mediated induction of cytotoxic effector cells capable of eliminating Epstein-Barr virus-transformed/immortalized lymphocytes in culture. J Natl Cancer Inst. 2001;93(22):1724–32. doi: 10.1093/jnci/93.22.1724 11717333

[39] Zhao B, Maruo S, Cooper A, C M R, Johannsen E, Kieff E, et al. RNAs induced by Epstein-Barr virus nuclear antigen 2 in lymphoblastoid cell lines. Proc Natl Acad Sci U S A. 2006;103(6):1900–5. doi: 10.1073/pnas.0510612103 16446431 PMC1413661

[40] Gregory CD, Rowe M, Rickinson AB. Different Epstein-Barr virus-B cell interactions in phenotypically distinct clones of a Burkitt’s lymphoma cell line. J Gen Virol. 1990;71 (Pt 7):1481–95. doi: 10.1099/0022-1317-71-7-1481 2165133

[41] Waldmann TA. The shared and contrasting roles of IL2 and IL15 in the life and death of normal and neoplastic lymphocytes: implications for cancer therapy. Cancer Immunol Res. 2015;3(3):219–27. doi: 10.1158/2326-6066.CIR-15-0009 25736261 PMC4351780

[42] Mitra B, Beri NR, Guo R, Burton EM, Murray-Nerger LA, Gewurz BE. Characterization of target gene regulation by the two Epstein-Barr virus oncogene LMP1 domains essential for B-cell transformation. mBio. 2023:e0233823. doi: 10.1128/mbio.02338-23 38009935 PMC10746160

[43] Teglund S, McKay C, Schuetz E, van Deursen JM, Stravopodis D, Wang D, et al. Stat5a and Stat5b proteins have essential and nonessential, or redundant, roles in cytokine responses. Cell. 1998;93(5):841–50. doi: 10.1016/s0092-8674(00)81444-0 9630227

[44] Kim E, Ilic N, Shrestha Y, Zou L, Kamburov A, Zhu C, et al. Systematic Functional Interrogation of Rare Cancer Variants Identifies Oncogenic Alleles. Cancer Discov. 2016;6(7):714–26. doi: 10.1158/2159-8290.CD-16-0160 27147599 PMC4930723

[45] Bromberg JF, Wrzeszczynska MH, Devgan G, Zhao Y, Pestell RG, Albanese C, et al. Stat3 as an oncogene. Cell. 1999;98(3):295–303. doi: 10.1016/s0092-8674(00)81959-5 10458605

[46] Dalton T, Doubrovina E, Pankov D, Reynolds R, Scholze H, Selvakumar A, et al. Epigenetic reprogramming sensitizes immunologically silent EBV+ lymphomas to virus-directed immunotherapy. Blood. 2020;135(21):1870–81. doi: 10.1182/blood.2019004126 32157281 PMC7243148

[47] Masucci MG, Contreras-Salazar B, Ragnar E, Falk K, Minarovits J, Ernberg I, et al. 5-Azacytidine up regulates the expression of Epstein-Barr virus nuclear antigen 2 (EBNA-2) through EBNA-6 and latent membrane protein in the Burkitt’s lymphoma line rael. J Virol. 1989;63(7):3135–41. 2470924 10.1128/jvi.63.7.3135-3141.1989 PMC250871

[48] Robertson KD, Manns A, Swinnen LJ, Zong JC, Gulley ML, Ambinder RF. CpG methylation of the major Epstein-Barr virus latency promoter in Burkitt’s lymphoma and Hodgkin’s disease. Blood. 1996;88(8):3129–36. 8874213

[49] Salamon D, Takacs M, Ujvari D, Uhlig J, Wolf H, Minarovits J, et al. Protein-DNA binding and CpG methylation at nucleotide resolution of latency-associated promoters Qp, Cp, and LMP1p of Epstein-Barr virus. J Virol. 2001;75(6):2584–96. doi: 10.1128/JVI.75.6.2584-2596.2001 11222681 PMC115881

[50] Guo R, Liang JH, Zhang Y, Lutchenkov M, Li Z, Wang Y, et al. Methionine Metabolism Controls the B-cell EBV Epigenome and Viral Latency. bioRxiv. 2022:2022.02.24.481783. 10.1016/j.cmet.2022.08.008 PMC9482757 36070681

[51] Alazard N, Gruffat H, Hiriart E, Sergeant A, Manet E. Differential hyperacetylation of histones H3 and H4 upon promoter-specific recruitment of EBNA2 in Epstein-Barr virus chromatin. J Virol. 2003;77(14):8166–72. doi: 10.1128/jvi.77.14.8166-8172.2003 12829856 PMC161941

[52] Lieberman PM. Chromatin Structure of Epstein-Barr Virus Latent Episomes. Curr Top Microbiol Immunol. 2015;390(Pt 1):71–102. doi: 10.1007/978-3-319-22822-8_5 26424644

[53] Tempera I, Klichinsky M, Lieberman PM. EBV latency types adopt alternative chromatin conformations. PLoS Pathog. 2011;7(7):e1002180. doi: 10.1371/journal.ppat.1002180 21829357 PMC3145795

[54] Tempera I, Wiedmer A, Dheekollu J, Lieberman PM. CTCF prevents the epigenetic drift of EBV latency promoter Qp. PLoS Pathog. 2010;6(8):e1001048. doi: 10.1371/journal.ppat.1001048 20730088 PMC2921154

[55] Caruso LB, Guo R, Keith K, Madzo J, Maestri D, Boyle S, et al. The nuclear lamina binds the EBV genome during latency and regulates viral gene expression. PLoS Pathog. 2022;18(4):e1010400. doi: 10.1371/journal.ppat.1010400 35421198 PMC9009669

[56] Price AM, Messinger JE, Luftig MA. c-Myc Represses Transcription of Epstein-Barr Virus Latent Membrane Protein 1 Early after Primary B Cell Infection. J Virol. 2018;92(2). doi: 10.1128/JVI.01178-17 29118124 PMC5752943

[57] Styles CT, Bazot Q, Parker GA, White RE, Paschos K, Allday MJ. EBV epigenetically suppresses the B cell-to-plasma cell differentiation pathway while establishing long-term latency. PLoS Biol. 2017;15(8):e2001992. doi: 10.1371/journal.pbio.2001992 28771465 PMC5542390

[58] Chen H, Lee JM, Zong Y, Borowitz M, Ng MH, Ambinder RF, et al. Linkage between STAT regulation and Epstein-Barr virus gene expression in tumors. J Virol. 2001;75(6):2929–37. doi: 10.1128/JVI.75.6.2929-2937.2001 11222718 PMC115919

[59] Messeguer X, Escudero R, Farre D, Nunez O, Martinez J, Alba MM. PROMO: detection of known transcription regulatory elements using species-tailored searches. Bioinformatics. 2002;18(2):333–4. doi: 10.1093/bioinformatics/18.2.333 11847087

[60] Farre D, Roset R, Huerta M, Adsuara JE, Rosello L, Alba MM, et al. Identification of patterns in biological sequences at the ALGGEN server: PROMO and MALGEN. Nucleic Acids Res. 2003;31(13):3651–3. doi: 10.1093/nar/gkg605 12824386 PMC169011

[61] Chen X, Kamranvar SA, Masucci MG. Oxidative stress enables Epstein-Barr virus-induced B-cell transformation by posttranscriptional regulation of viral and cellular growth-promoting factors. Oncogene. 2016;35(29):3807–16. doi: 10.1038/onc.2015.450 26592445

[62] Li X, Bhaduri-McIntosh S. A Central Role for STAT3 in Gammaherpesvirus-Life Cycle and -Diseases. Front Microbiol. 2016;7:1052. doi: 10.3389/fmicb.2016.01052 27458446 PMC4937026

[63] Koganti S, de la Paz A, Freeman AF, Bhaduri-McIntosh S. B lymphocytes from patients with a hypomorphic mutation in STAT3 resist Epstein-Barr virus-driven cell proliferation. J Virol. 2014;88(1):516–24. doi: 10.1128/JVI.02601-13 24173212 PMC3911703

[64] Wang LW, Shen H, Nobre L, Ersing I, Paulo JA, Trudeau S, et al. Epstein-Barr-Virus-Induced One-Carbon Metabolism Drives B Cell Transformation. Cell Metab. 2019;30(3):539–55 e11. doi: 10.1016/j.cmet.2019.06.003 31257153 PMC6720460

[65] Wang F, Gregory CD, Rowe M, Rickinson AB, Wang D, Birkenbach M, et al. Epstein-Barr virus nuclear antigen 2 specifically induces expression of the B-cell activation antigen CD23. Proc Natl Acad Sci U S A. 1987;84(10):3452–6. doi: 10.1073/pnas.84.10.3452 3033649 PMC304889

[66] Aman P, Rowe M, Kai C, Finke J, Rymo L, Klein E, et al. Effect of the EBNA-2 gene on the surface antigen phenotype of transfected EBV-negative B-lymphoma lines. Int J Cancer. 1990;45(1):77–82. doi: 10.1002/ijc.2910450115 2153641

[67] Chen H, Lee JM, Wang Y, Huang DP, Ambinder RF, Hayward SD. The Epstein-Barr virus latency BamHI-Q promoter is positively regulated by STATs and Zta interference with JAK/STAT activation leads to loss of BamHI-Q promoter activity. Proc Natl Acad Sci U S A. 1999;96(16):9339–44. doi: 10.1073/pnas.96.16.9339 10430944 PMC17784

[68] Barbour H, Daou S, Hendzel M, Affar EB. Polycomb group-mediated histone H2A monoubiquitination in epigenome regulation and nuclear processes. Nat Commun. 2020;11(1):5947. doi: 10.1038/s41467-020-19722-9 33230107 PMC7683540

[69] Lu F, Wiedmer A, Martin KA, Wickramasinghe P, Kossenkov AV, Lieberman PM. Coordinate Regulation of TET2 and EBNA2 Controls the DNA Methylation State of Latent Epstein-Barr Virus. J Virol. 2017;91(20). doi: 10.1128/JVI.00804-17 28794029 PMC5625499

[70] Wille CK, Li Y, Rui L, Johannsen EC, Kenney SC. Restricted TET2 Expression in Germinal Center Type B Cells Promotes Stringent Epstein-Barr Virus Latency. J Virol. 2017;91(5). doi: 10.1128/JVI.01987-16 28003489 PMC5309966

[71] Gires O, Kohlhuber F, Kilger E, Baumann M, Kieser A, Kaiser C, et al. Latent membrane protein 1 of Epstein-Barr virus interacts with JAK3 and activates STAT proteins. EMBO J. 1999;18(11):3064–73. doi: 10.1093/emboj/18.11.3064 10357818 PMC1171388

[72] Brennan P, Floettmann JE, Mehl A, Jones M, Rowe M. Mechanism of action of a novel latent membrane protein-1 dominant negative. J Biol Chem. 2001;276(2):1195–203. doi: 10.1074/jbc.M005461200 11031256

[73] Higuchi M, Kieff E, Izumi KM. The Epstein-Barr virus latent membrane protein 1 putative Janus kinase 3 (JAK3) binding domain does not mediate JAK3 association or activation in B-lymphoma or lymphoblastoid cell lines. J Virol. 2002;76(1):455–9. doi: 10.1128/jvi.76.1.455-459.2002 11739714 PMC135721

[74] Lambert SL, Martinez OM. Latent membrane protein 1 of EBV activates phosphatidylinositol 3-kinase to induce production of IL-10. J Immunol. 2007;179(12):8225–34. doi: 10.4049/jimmunol.179.12.8225 18056366

[75] Minamitani T, Ma Y, Zhou H, Kida H, Tsai CY, Obana M, et al. Mouse model of Epstein-Barr virus LMP1- and LMP2A-driven germinal center B-cell lymphoproliferative disease. Proc Natl Acad Sci U S A. 2017;114(18):4751–6. doi: 10.1073/pnas.1701836114 28351978 PMC5422827

[76] Du T, Zhou G, Roizman B. Modulation of reactivation of latent herpes simplex virus 1 in ganglionic organ cultures by p300/CBP and STAT3. Proc Natl Acad Sci U S A. 2013;110(28):E2621–8. doi: 10.1073/pnas.1309906110 23788661 PMC3710862

[77] Hill ER, Koganti S, Zhi J, Megyola C, Freeman AF, Palendira U, et al. Signal transducer and activator of transcription 3 limits Epstein-Barr virus lytic activation in B lymphocytes. J Virol. 2013;87(21):11438–46. doi: 10.1128/JVI.01762-13 23966384 PMC3807321

[78] King CA, Li X, Barbachano-Guerrero A, Bhaduri-McIntosh S. STAT3 Regulates Lytic Activation of Kaposi’s Sarcoma-Associated Herpesvirus. J Virol. 2015;89(22):11347–55. doi: 10.1128/JVI.02008-15 26339061 PMC4645641

[79] Li X, Burton EM, Koganti S, Zhi J, Doyle F, Tenenbaum SA, et al. KRAB-ZFP Repressors Enforce Quiescence of Oncogenic Human Herpesviruses. J Virol. 2018;92(14). doi: 10.1128/JVI.00298-18 29695433 PMC6026741

[80] Shair KH, Bendt KM, Edwards RH, Bedford EC, Nielsen JN, Raab-Traub N. EBV latent membrane protein 1 activates Akt, NFkappaB, and Stat3 in B cell lymphomas. PLoS Pathog. 2007;3(11):e166. doi: 10.1371/journal.ppat.0030166 17997602 PMC2065877

[81] Shair KH, Raab-Traub N. Transcriptome changes induced by Epstein-Barr virus LMP1 and LMP2A in transgenic lymphocytes and lymphoma. mBio. 2012;3(5). doi: 10.1128/mBio.00288-12 22991431 PMC3448168

[82] Frontzek F, Staiger AM, Wullenkord R, Grau M, Zapukhlyak M, Kurz KS, et al. Molecular profiling of EBV associated diffuse large B-cell lymphoma. Leukemia. 2023;37(3):670–9. doi: 10.1038/s41375-022-01804-w 36604606 PMC9991915

[83] Butzmann A, Sridhar K, Jangam D, Song H, Singh A, Kumar J, et al. Mutations in JAK/STAT and NOTCH1 Genes Are Enriched in Post-Transplant Lymphoproliferative Disorders. Front Oncol. 2021;11:790481. doi: 10.3389/fonc.2021.790481 35111674 PMC8801788

[84] Leeman-Neill RJ, Soderquist CR, Montanari F, Raciti P, Park D, Radeski D, et al. Phenogenomic heterogeneity of post-transplant plasmablastic lymphomas. Haematologica. 2022;107(1):201–10. doi: 10.3324/haematol.2020.267294 33297669 PMC8719101

[85] Vaysberg M, Lambert SL, Krams SM, Martinez OM. Activation of the JAK/STAT pathway in Epstein Barr virus+-associated posttransplant lymphoproliferative disease: role of interferon-gamma. Am J Transplant. 2009;9(10):2292–302. doi: 10.1111/j.1600-6143.2009.02781.x 19656130 PMC2774223

[86] Garcia-Reyero J, Martinez Magunacelaya N, Gonzalez de Villambrosia S, Loghavi S, Gomez Mediavilla A, Tonda R, et al. Genetic lesions in MYC and STAT3 drive oncogenic transcription factor overexpression in plasmablastic lymphoma. Haematologica. 2021;106(4):1120–8. doi: 10.3324/haematol.2020.251579 32273478 PMC8018103

[87] Yang Z, Xiang Q, Nicholas J. Direct and biologically significant interactions of human herpesvirus 8 interferon regulatory factor 1 with STAT3 and Janus kinase TYK2. PLoS Pathog. 2023;19(11):e1011806. doi: 10.1371/journal.ppat.1011806 37983265 PMC10695398

[88] Rivera-Soto R, Dissinger NJ, Damania B. Kaposi’s Sarcoma-Associated Herpesvirus Viral Interleukin-6 Signaling Upregulates Integrin beta3 Levels and Is Dependent on STAT3. J Virol. 2020;94(5). 10.1128/JVI.01384-19 PMC7022358 31801855

[89] Ramalingam D, Ziegelbauer JM. Viral microRNAs Target a Gene Network, Inhibit STAT Activation, and Suppress Interferon Responses. Sci Rep. 2017;7:40813. doi: 10.1038/srep40813 28102325 PMC5244407

[90] Lee MS, Jones T, Song DY, Jang JH, Jung JU, Gao SJ. Exploitation of the complement system by oncogenic Kaposi’s sarcoma-associated herpesvirus for cell survival and persistent infection. PLoS Pathog. 2014;10(9):e1004412. doi: 10.1371/journal.ppat.1004412 25254972 PMC4177982

[91] Liu X, Sadaoka T, Krogmann T, Cohen JI. Epstein-Barr Virus (EBV) Tegument Protein BGLF2 Suppresses Type I Interferon Signaling To Promote EBV Reactivation. J Virol. 2020;94(11). doi: 10.1128/JVI.00258-20 32213613 PMC7269453

[92] Reddy SS, Foreman HC, Sioux TO, Park GH, Poli V, Reich NC, et al. Ablation of STAT3 in the B Cell Compartment Restricts Gammaherpesvirus Latency In Vivo. mBio. 2016;7(4). 10.1128/mBio.00723-16 PMC4981709 27486189

[93] Hogan CH, Owens SM, Reynoso GV, Liao Y, Meyer TJ, Zelazowska MA, et al. Multifaceted roles for STAT3 in gammaherpesvirus latency revealed through in vivo B cell knockout models. mBio. 2024;15(2):e0299823. doi: 10.1128/mbio.02998-23 38170993 PMC10870824

[94] Fike AJ, Chodisetti SB, Wright NE, Bricker KN, Domeier PP, Maienschein-Cline M, et al. STAT3 signaling in B cells controls germinal center zone organization and recycling. Cell Rep. 2023;42(5):112512. doi: 10.1016/j.celrep.2023.112512 37200190 PMC10311431

[95] McAtee CL, Lubega J, Underbrink K, Curry K, Msaouel P, Barrow M, et al. Association of Rituximab Use With Adverse Events in Children, Adolescents, and Young Adults. JAMA Netw Open. 2021;4(2):e2036321. doi: 10.1001/jamanetworkopen.2020.36321 33533931 PMC7859842

[96] Kasi PM, Tawbi HA, Oddis CV, Kulkarni HS. Clinical review: Serious adverse events associated with the use of rituximab—a critical care perspective. Crit Care. 2012;16(4):231. doi: 10.1186/cc11304 22967460 PMC3580676

[97] Zeiser R, von Bubnoff N, Butler J, Mohty M, Niederwieser D, Or R, et al. Ruxolitinib for Glucocorticoid-Refractory Acute Graft-versus-Host Disease. N Engl J Med. 2020;382(19):1800–10. doi: 10.1056/NEJMoa1917635 32320566

[98] Zhang B, Kracker S, Yasuda T, Casola S, Vanneman M, Homig-Holzel C, et al. Immune surveillance and therapy of lymphomas driven by Epstein-Barr virus protein LMP1 in a mouse model. Cell. 2012;148(4):739–51. doi: 10.1016/j.cell.2011.12.031 22341446 PMC3313622

[99] Taylor GS, Long HM, Brooks JM, Rickinson AB, Hislop AD. The immunology of Epstein-Barr virus-induced disease. Annu Rev Immunol. 2015;33:787–821. doi: 10.1146/annurev-immunol-032414-112326 25706097

[100] Choi IK, Wang Z, Ke Q, Hong M, Paul DW, Fernandes SM, et al. Mechanism of EBV inducing anti-tumour immunity and its therapeutic use. Nature. 2021;590(7844):157–62. doi: 10.1038/s41586-020-03075-w 33361812 PMC7864874

[101] Spolski R, Leonard WJ. Interleukin-21: basic biology and implications for cancer and autoimmunity. Annu Rev Immunol. 2008;26:57–79. doi: 10.1146/annurev.immunol.26.021607.090316 17953510

[102] Skak K, Kragh M, Hausman D, Smyth MJ, Sivakumar PV. Interleukin 21: combination strategies for cancer therapy. Nat Rev Drug Discov. 2008;7(3):231–40. doi: 10.1038/nrd2482 18259184

[103] Long D, Chen Y, Wu H, Zhao M, Lu Q. Clinical significance and immunobiology of IL-21 in autoimmunity. J Autoimmun. 2019;99:1–14. doi: 10.1016/j.jaut.2019.01.013 30773373

[104] O’Reilly RJ, Prockop S, Oved JH. Virus-specific T-cells from third party or transplant donors for treatment of EBV lymphoproliferative diseases arising post hematopoietic cell or solid organ transplantation. Front Immunol. 2023;14:1290059. doi: 10.3389/fimmu.2023.1290059 38274824 PMC10808771

[105] Deng S, Sun Z, Qiao J, Liang Y, Liu L, Dong C, et al. Targeting tumors with IL-21 reshapes the tumor microenvironment by proliferating PD-1intTim-3-CD8+ T cells. JCI Insight. 2020;5(7). doi: 10.1172/jci.insight.132000 32271164 PMC7205272

[106] Schmidt H, Brown J, Mouritzen U, Selby P, Fode K, Svane IM, et al. Safety and clinical effect of subcutaneous human interleukin-21 in patients with metastatic melanoma or renal cell carcinoma: a phase I trial. Clin Cancer Res. 2010;16(21):5312–9. doi: 10.1158/1078-0432.CCR-10-1809 20959407

[107] Ahmed M, Lopez-Albaitero A, Pankov D, Santich BH, Liu H, Yan S, et al. TCR-mimic bispecific antibodies targeting LMP2A show potent activity against EBV malignancies. JCI Insight. 2018;3(4). doi: 10.1172/jci.insight.97805 29467338 PMC5916246

[108] Martin E, Winter S, Garcin C, Tanita K, Hoshino A, Lenoir C, et al. Role of IL-27 in Epstein-Barr virus infection revealed by IL-27RA deficiency. Nature. 2024;628(8008):620–9. doi: 10.1038/s41586-024-07213-6 38509369

[109] Greenfeld H, Takasaki K, Walsh MJ, Ersing I, Bernhardt K, Ma Y, et al. TRAF1 Coordinates Polyubiquitin Signaling to Enhance Epstein-Barr Virus LMP1-Mediated Growth and Survival Pathway Activation. PLoS Pathog. 2015;11(5):e1004890. doi: 10.1371/journal.ppat.1004890 25996949 PMC4440769

[110] Ma Y, Walsh MJ, Bernhardt K, Ashbaugh CW, Trudeau SJ, Ashbaugh IY, et al. CRISPR/Cas9 Screens Reveal Epstein-Barr Virus-Transformed B Cell Host Dependency Factors. Cell Host Microbe. 2017;21(5):580–91 e7. doi: 10.1016/j.chom.2017.04.005 28494239 PMC8938989

[111] Jiang S, Wang LW, Walsh MJ, Trudeau SJ, Gerdt C, Zhao B, et al. CRISPR/Cas9-Mediated Genome Editing in Epstein-Barr Virus-Transformed Lymphoblastoid B-Cell Lines. Curr Protoc Mol Biol. 2018;121:31 12 1–31 12 23. doi: 10.1002/cpmb.51 29337376

[112] Sanson KR, Hanna RE, Hegde M, Donovan KF, Strand C, Sullender ME, et al. Optimized libraries for CRISPR-Cas9 genetic screens with multiple modalities. Nat Commun. 2018;9(1):5416. doi: 10.1038/s41467-018-07901-8 30575746 PMC6303322

[113] Yiu SPT, Guo R, Zerbe C, Weekes MP, Gewurz BE. Epstein-Barr virus BNRF1 destabilizes SMC5/6 cohesin complexes to evade its restriction of replication compartments. Cell Rep. 2022;38(10):110411. doi: 10.1016/j.celrep.2022.110411 35263599 PMC8981113

[114] Guo R, Liang JH, Zhang Y, Lutchenkov M, Li Z, Wang Y, et al. Methionine metabolism controls the B cell EBV epigenome and viral latency. Cell Metab. 2022;34(9):1280–97 e9. doi: 10.1016/j.cmet.2022.08.008 36070681 PMC9482757

[115] Love MI, Huber W, Anders S. Moderated estimation of fold change and dispersion for RNA-seq data with DESeq2. Genome Biol. 2014;15(12):550. doi: 10.1186/s13059-014-0550-8 25516281 PMC4302049

[116] Cunningham F, Allen JE, Allen J, Alvarez-Jarreta J, Amode MR, Armean IM, et al. Ensembl 2022. Nucleic Acids Res. 2022;50(D1):D988–D95. doi: 10.1093/nar/gkab1049 34791404 PMC8728283

