# Supplementary figures and images for: Germinal center cytokine driven epigenetic control of Epstein-Barr virus latency gene expression
Source: PLoS Pathog. 2024 Apr 29;20(4):e1011939. doi: 10.1371/journal.ppat.1011939 (PMC11081508; doi:10.1371/journal.ppat.1011939)

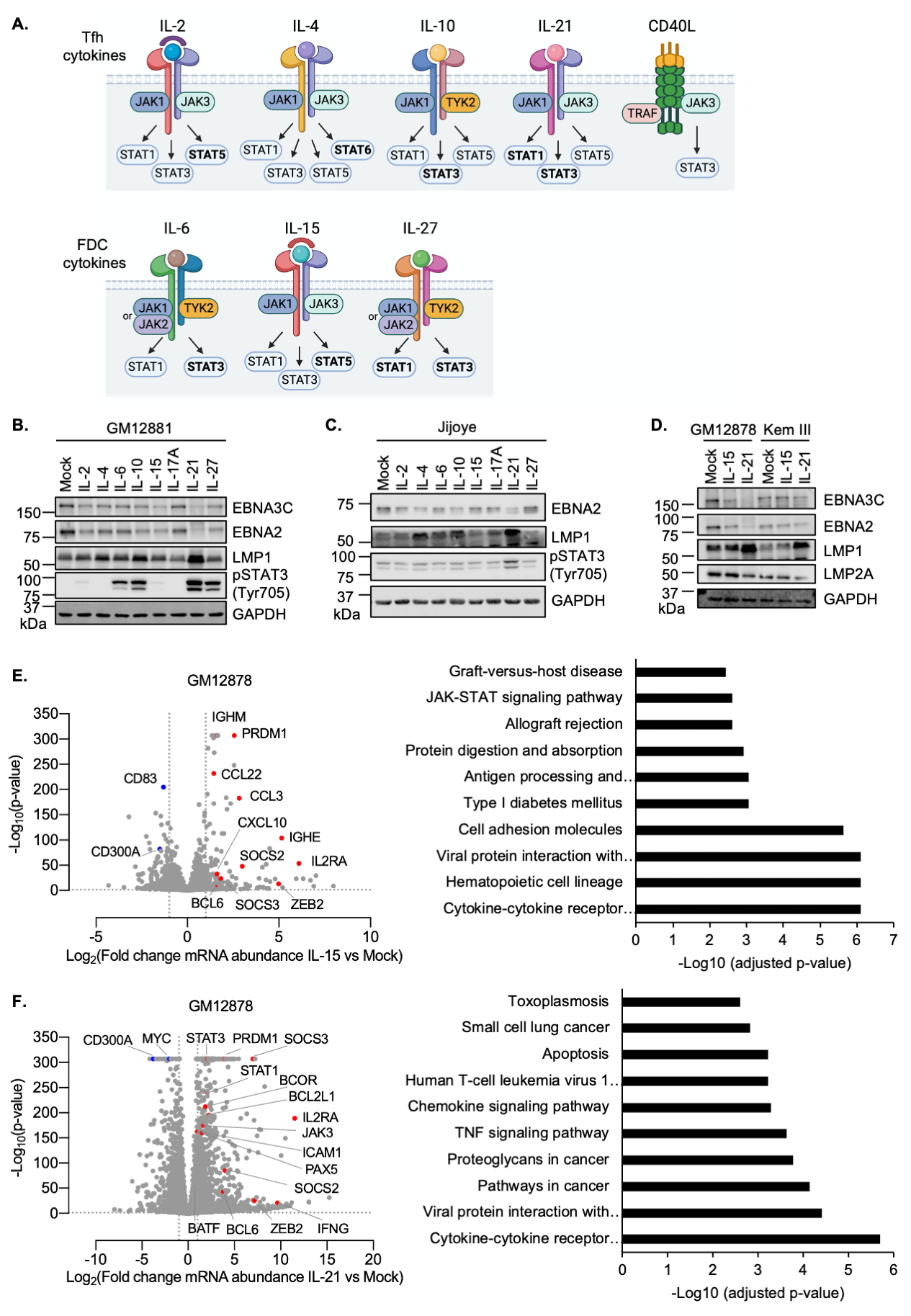

Supplement: S1 Fig — (A) Schematic of Tfh and FDC cytokine driven JAK/STAT signaling. (BioRender was used to create the schematic models.) (B-C) Immunoblot analysis of WCL from GM12881 (B) and latency III Jijoye (C) cells treated with the indicated cytokines for six days. (D) Immunoblot analysis of WCL from GM12878 and Kem III cells six days post mock, IL-15 or IL-21 treatment. (E) Volcano plot (left) and KEGG pathway analysis (right) of host genes expression in GM12878 stimulated by IL-15 versus mock-simulated for six days from n = 3 independent replicates. The top 10 most differentially expressed KEGG pathways are shown. (F) Volcano plot (left) and KEGG pathway analysis (right) of host genes expression in GM12878 stimulated by IL-21 versus mock-simulated for six days from n = 3 independent replicates. Cytokines were used at 100 ng/ml and were refreshed every two days. Immunoblots are representative of n = 3 replicates. (TIFF) [file ppat.1011939.s001.tiff]

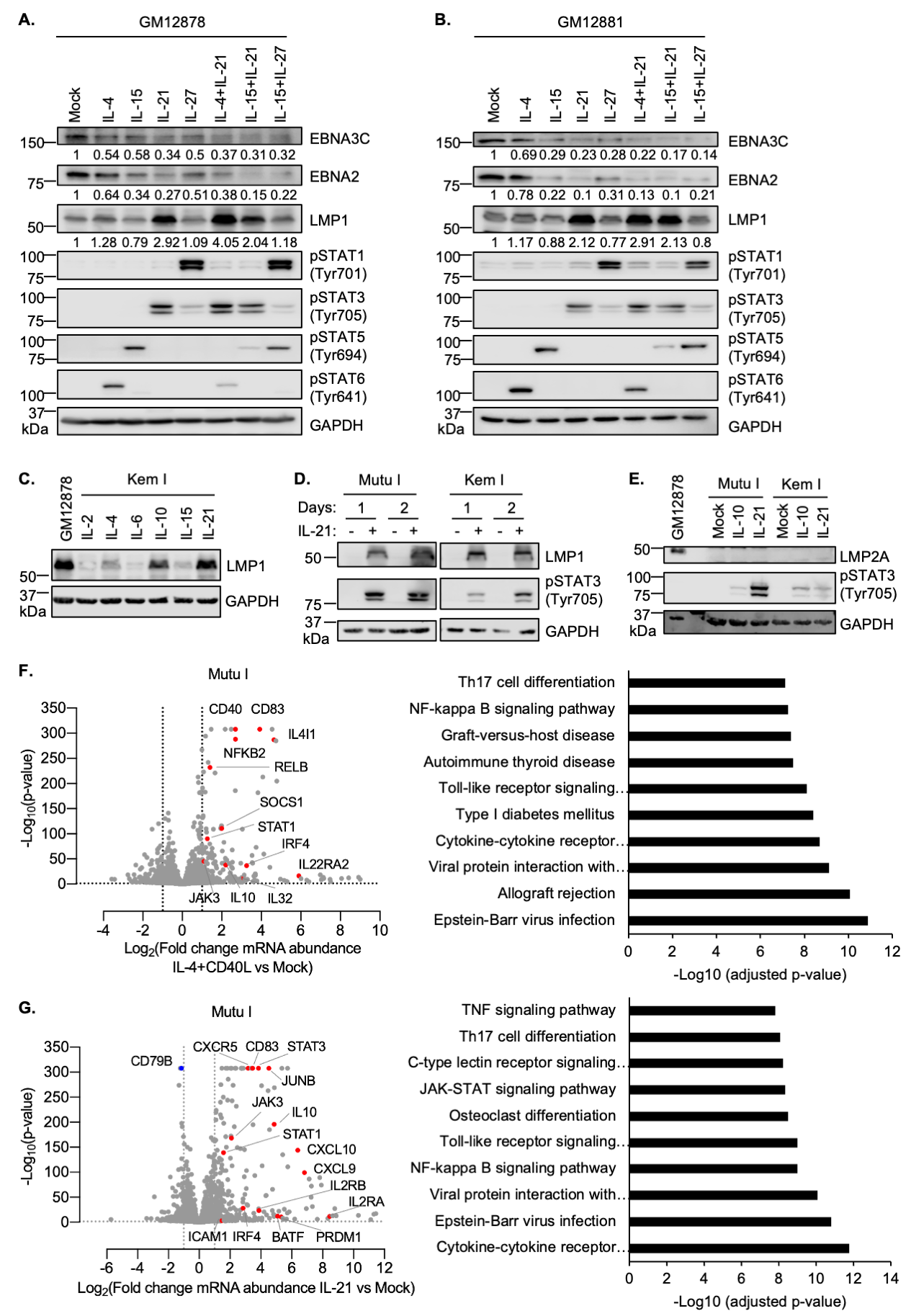

Supplement: S2 Fig — (A-B) Immunoblot analysis of WCL from GM12878 (A) or GM12881 (B) LCLs that were mock treated or treated with the indicated cytokines for six days. Densitometry values of GAPDH normalized EBNA3C, EBNA2 and LMP1 levels are shown beneath each row. (C) Immunoblot analysis of WCL from latency I Kem I Burkitt B cells treated with the indicated cytokines for 24 hours. (D) Immunoblot analysis of WCL from Mutu I and Kem I treated with IL-21 for one or two days, as indicated. (E) Immunoblot analysis of WCL from Mutu I and Kem I one day post mock, IL-10 or IL-21 treatment. GM12878 WCL was included as a positive control. (F) Volcano plot (left) and KEGG pathway analysis (right) of differentially expressed Mutu I host genes one day after IL-4+CD40L vs mock stimulation from n = 3 independent replicates. (G) Volcano plot (left) and KEGG pathway analysis (right) of differentially expressed Mutu I host genes one day after IL-4+CD40L vs mock stimulation from n = 3 independent replicates. The top 10 KEGG pathways amongst differentially regulated genes are shown. Cytokines and CD40L were used at 100 ng/ml and 50 ng/ml for EBV latency III and I cells, respectively. Immunoblots are representative of n = 3 replicates. (TIFF) [file ppat.1011939.s002.tiff]

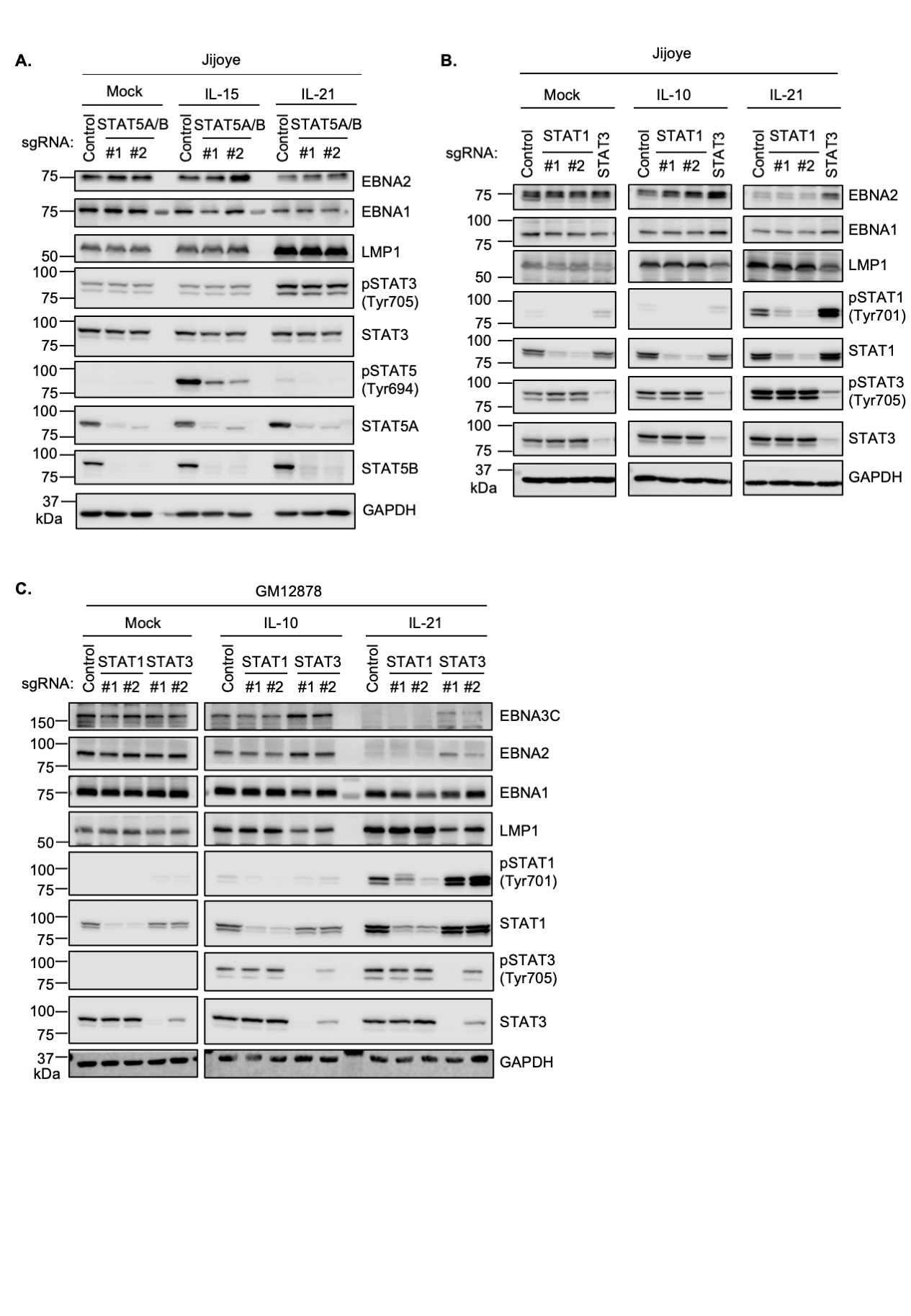

Supplement: S3 Fig — (A) Immunoblot analysis of WCL from latency III Jijoye B cells expressing control sgRNA or sgRNA targeting STAT5A and STAT5B, mock treated or treated with IL-15 or IL-21 for six days. (B-C) Immunoblot analysis of WCL from Jijoye (B) or GM12878 (C) expressing control sgRNA or sgRNA targeting the indicated STAT transcription factor gene, mock treated or treated with IL-10 or IL-21 for six days. Blots are representative of n = 3 replicates. Cytokines were used at 100 ng/ml and refreshed every 2 days. (TIFF) [file ppat.1011939.s003.tiff]

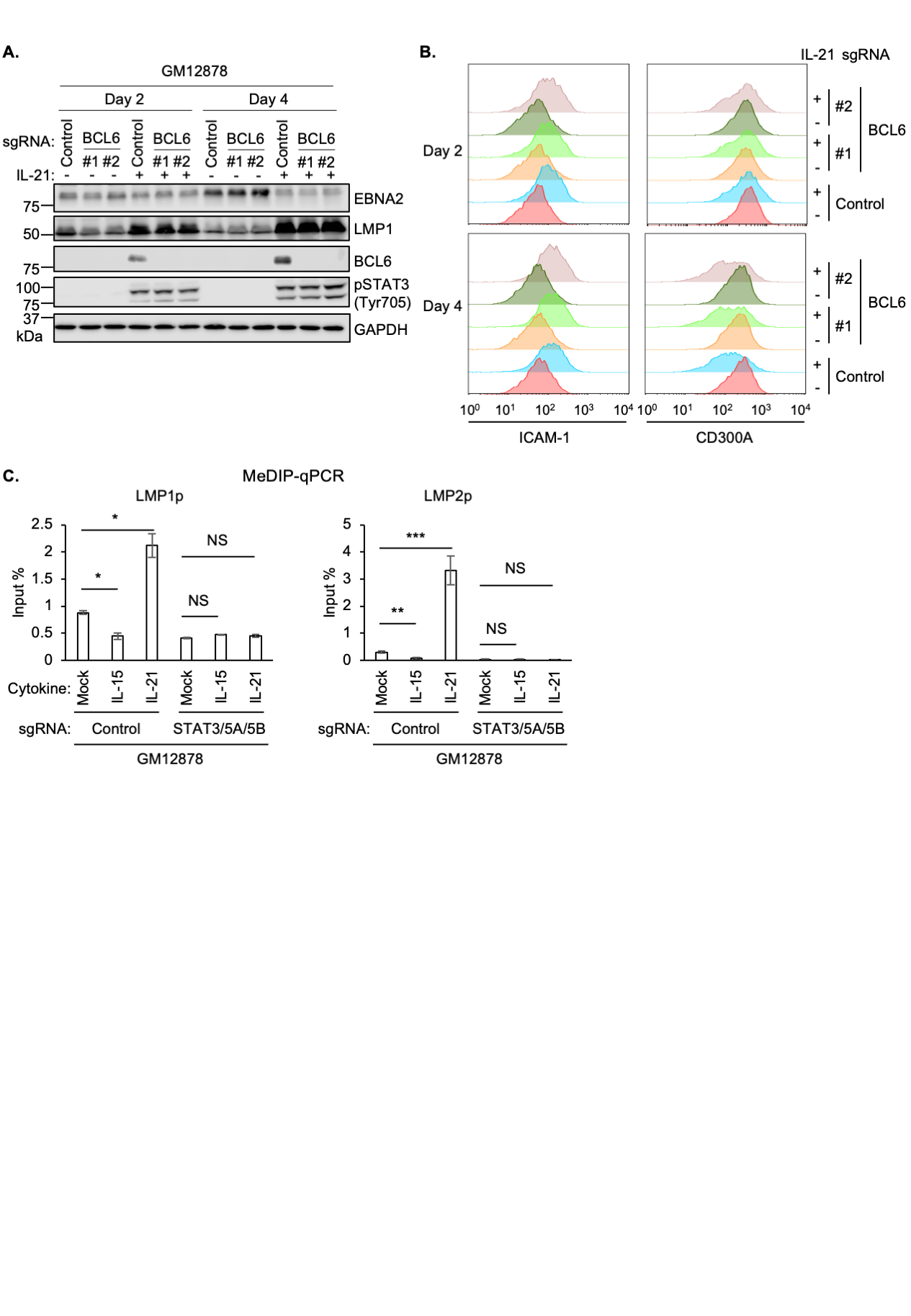

Supplement: S4 Fig — (A) Immunoblot analysis of WCL from GM12878 cells expressing control sgRNA or independent BCL6 targeting sgRNA that were mock treated or treated with IL-21 (100 ng/ml) for two or four days. Blot is representative of n = 3 replicates. (B) Flow cytometry analysis of LMP1 target ICAM-1 and EBNA2 target CD300A plasma membrane expression in GM12878 expressing control sgRNA or BCL6 sgRNA and mock treated or IL-21 treated for 2 or 4 days, as indicated. (C) MeDIP-qPCR analysis of GM12878 expressing control or sgRNA targeting STAT3/5A/5B, mock treated or treated with IL-15 or IL-21 for six days, followed by qPCR with primers targeting the LMP1 promoter (LMP1p, left) or LMP2 promoter (LMP2p, right). Mean ± SD ChIP-qPCR % input values from n = 3 replicates are shown. *p < 0.05; **p < 0.01; ***p < 0.001. (TIFF) [file ppat.1011939.s004.tiff]

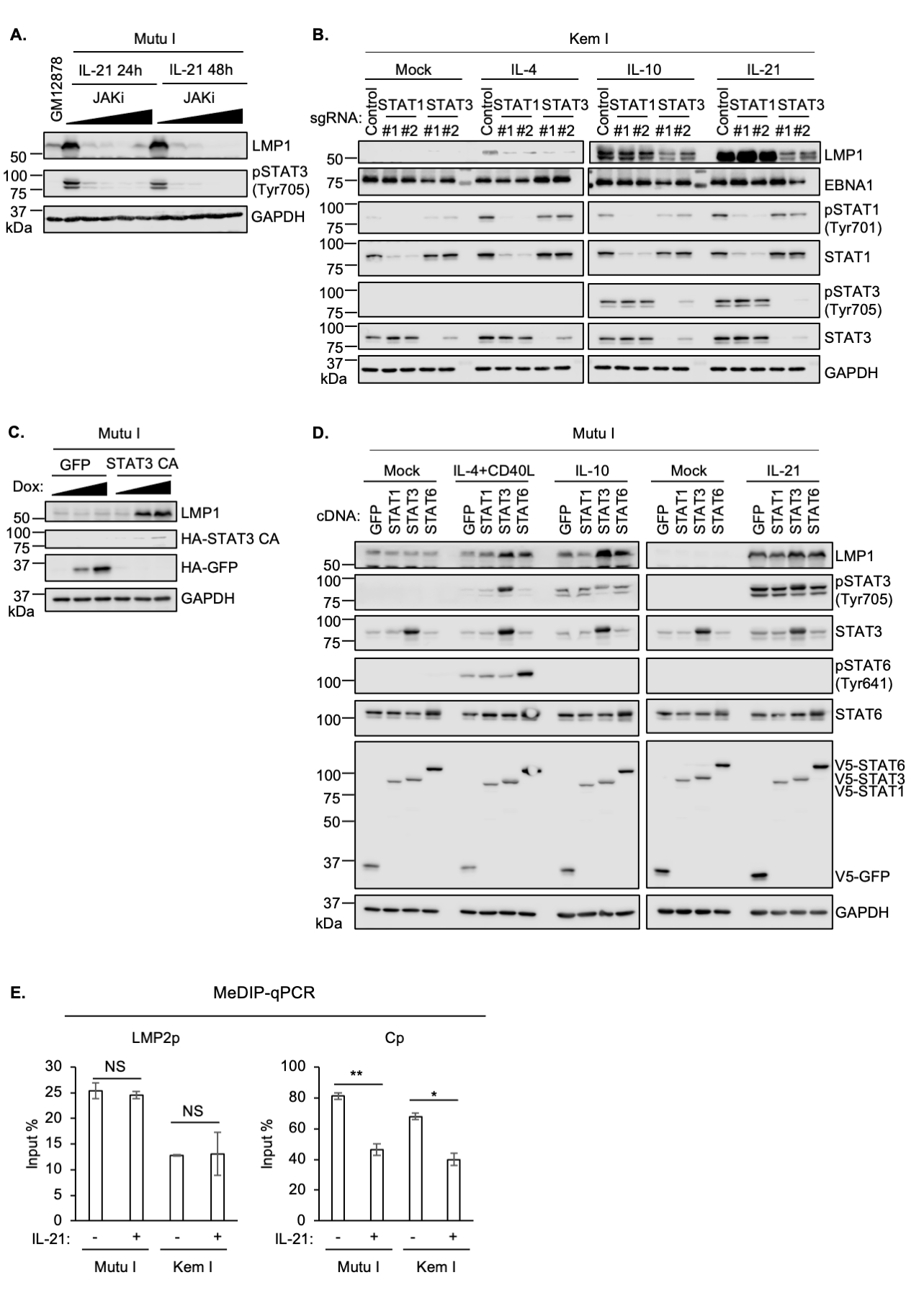

Supplement: S5 Fig — (A) Immunoblot analysis of WCL from Mutu I cells treated with JAKi (0–1,000 ng/ml) for one hour, followed by IL-21 treatment for one or two days. GM12878 cell lysate was included as a positive control. (B) Immunoblot analysis of WCL from Kem I expressing control sgRNA or sgRNA targeting STAT1 or STAT3, mock treated or treated with the indicated cytokine for one day. (C) Immunoblot analysis of WCL from Mutu I conditionally induced for control GFP or constitutively active STAT3 for one day by 0.5 or 1 μg/ml doxycycline. (D) Immunoblot analysis of Mutu I expressing the indicated control GFP or STAT cDNA and stimulated as indicated for 1 day. (E) MeDIP-qPCR of the LMP2 promoter (left) and C promoter (right) in Mutu I and Kem I, mock treated or IL-21 treated for one day. Mean ± SD input % of n = 3 replicates are shown, *p < 0.05; **p < 0.01. All cytokines were used at 50 ng/ml. Blots are representative of n = 3 replicates. (TIFF) [file ppat.1011939.s005.tiff]

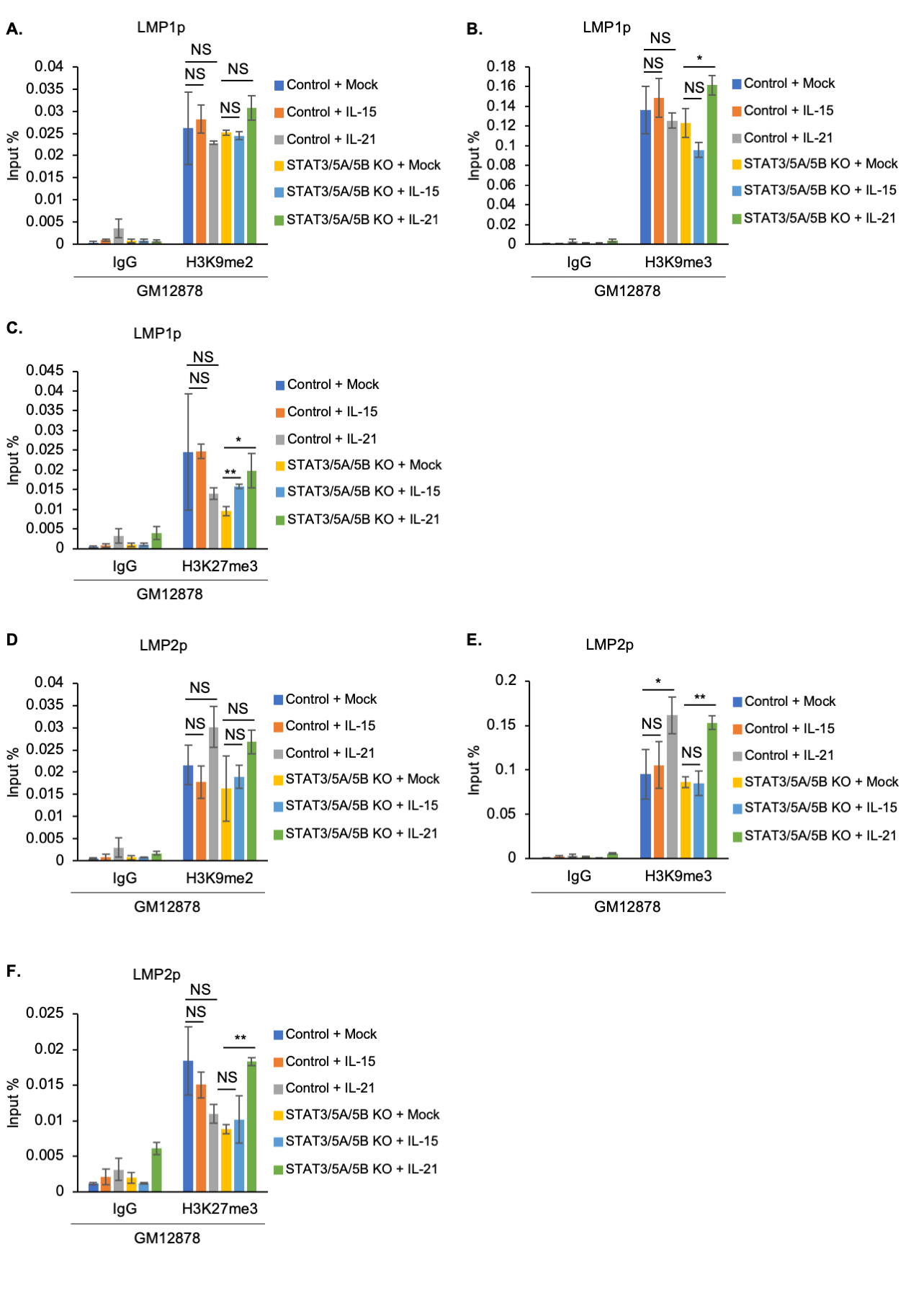

Supplement: S6 Fig — (A-C) ChIP-qPCR analysis of LMP1 promoter H3K9me2 (A), H3K9me3 (B) or H3K27me3 (C) abundances from GM12878 expressing control or STAT3/5A/5B targeting sgRNAs, mock treated or treated with 100ng/ml IL-15 or IL-21 for six days. (D-F) ChIP-qPCR analysis of LMP2 promoter H3K9me2 (D), H3K9me3 (E) or H3K27me3 (F) abundances in GM12878 expressing control or STAT3/5A/5B targeting sgRNAs, mock treated or treated with IL-15 or IL-21 for six days. Mean ± SD input % of n = 3 replicates are shown, *p < 0.05; **p < 0.01. (TIFF) [file ppat.1011939.s006.tiff]

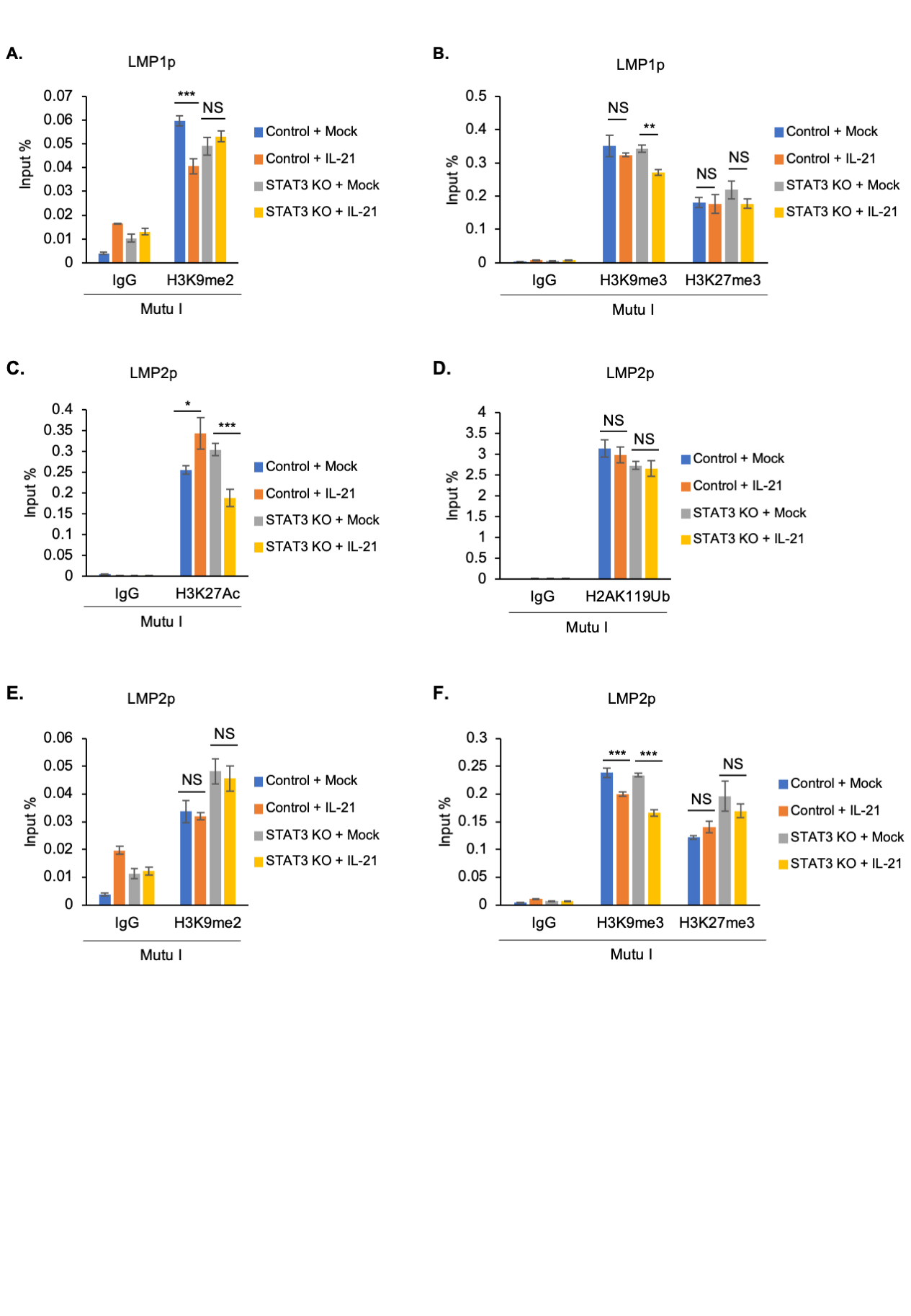

Supplement: S7 Fig — (A-B) ChIP-qPCR analysis of LMP1 promoter H3K9me2 (A) or H3K9me3 and H3K27me3 (B) abundances from Mutu I expressing control or STAT3 targeting sgRNA, mock treated or treated with IL-21. (C-F) ChIP-qPCR analysis of LMP2 promoter H3K27Ac (C) or H2AK119Ub (D), H3K9me2 (E) or H3K9me3 and H3K27me3 (F) abundances in Mutu I expressing control or STAT3 targeting sgRNAs, mock treated or treated with IL-21. Cells were treated with 50 ng/ml IL-21 for one day. Mean ± SD input % of n = 3 replicates are shown, *p < 0.05; **p < 0.01. (TIFF) [file ppat.1011939.s007.tiff]

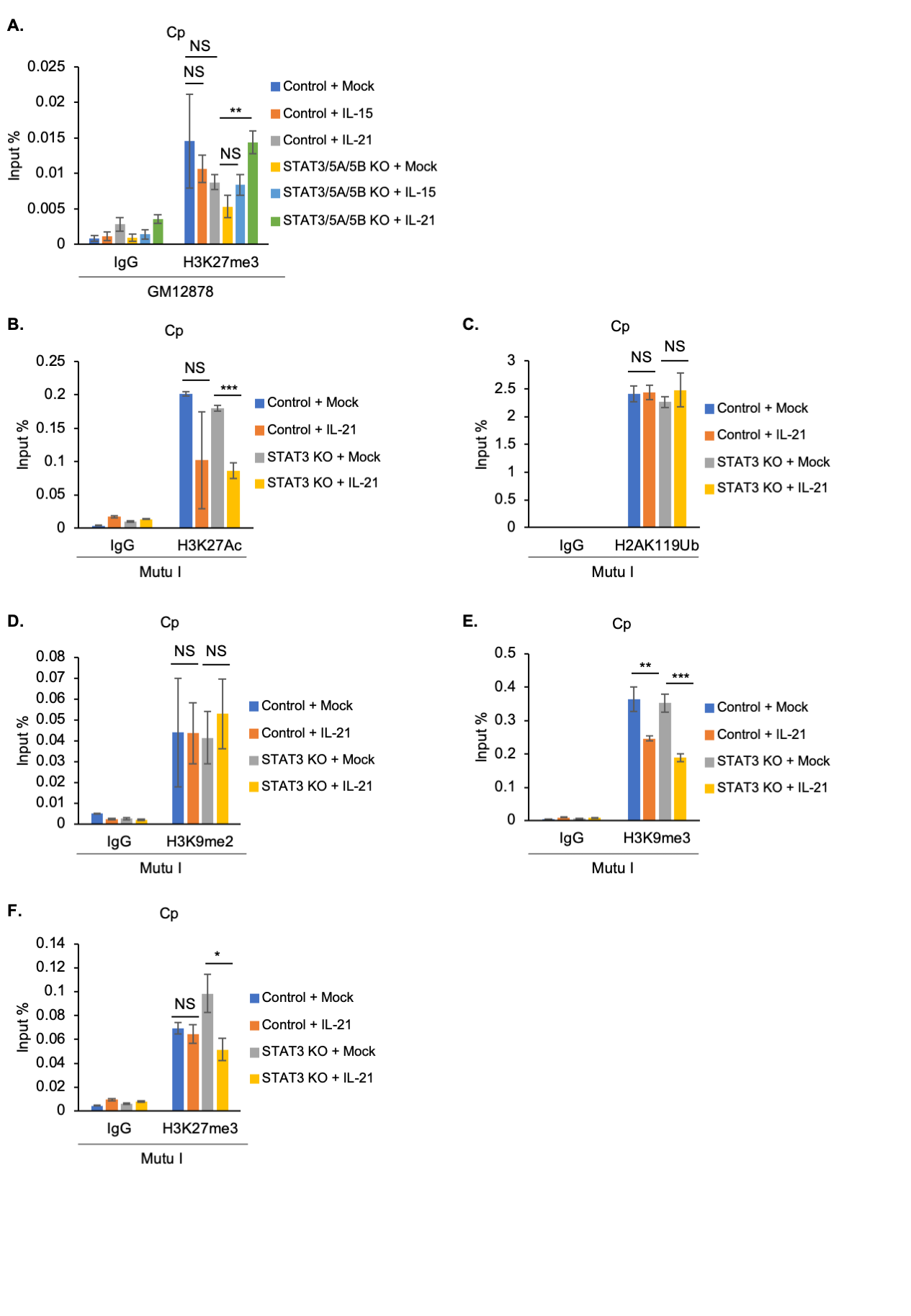

Supplement: S8 Fig — (A) ChIP-qPCR analysis of Cp H3K27me3 abundances in Mutu I expressing control or STAT3/5A/5B targeting sgRNAs, mock treated or treated with IL-15 or IL-21 (100ng/ml) for six days. (B-F) ChIP-qPCR analysis of Cp H3K27Ac (B), H2AK119Ub (C), H3K9me2 (D), H3K9me3 (E) or H3K27me3 (F) abundances in Mutu I expressing control or STAT3 targeting sgRNAs, mock treated or treated with IL-21 50 ng/ml for 1 day. Mean ± SD input % of n = 3 replicates are shown, *p < 0.05; **p < 0.01. (TIFF) [file ppat.1011939.s008.tiff]

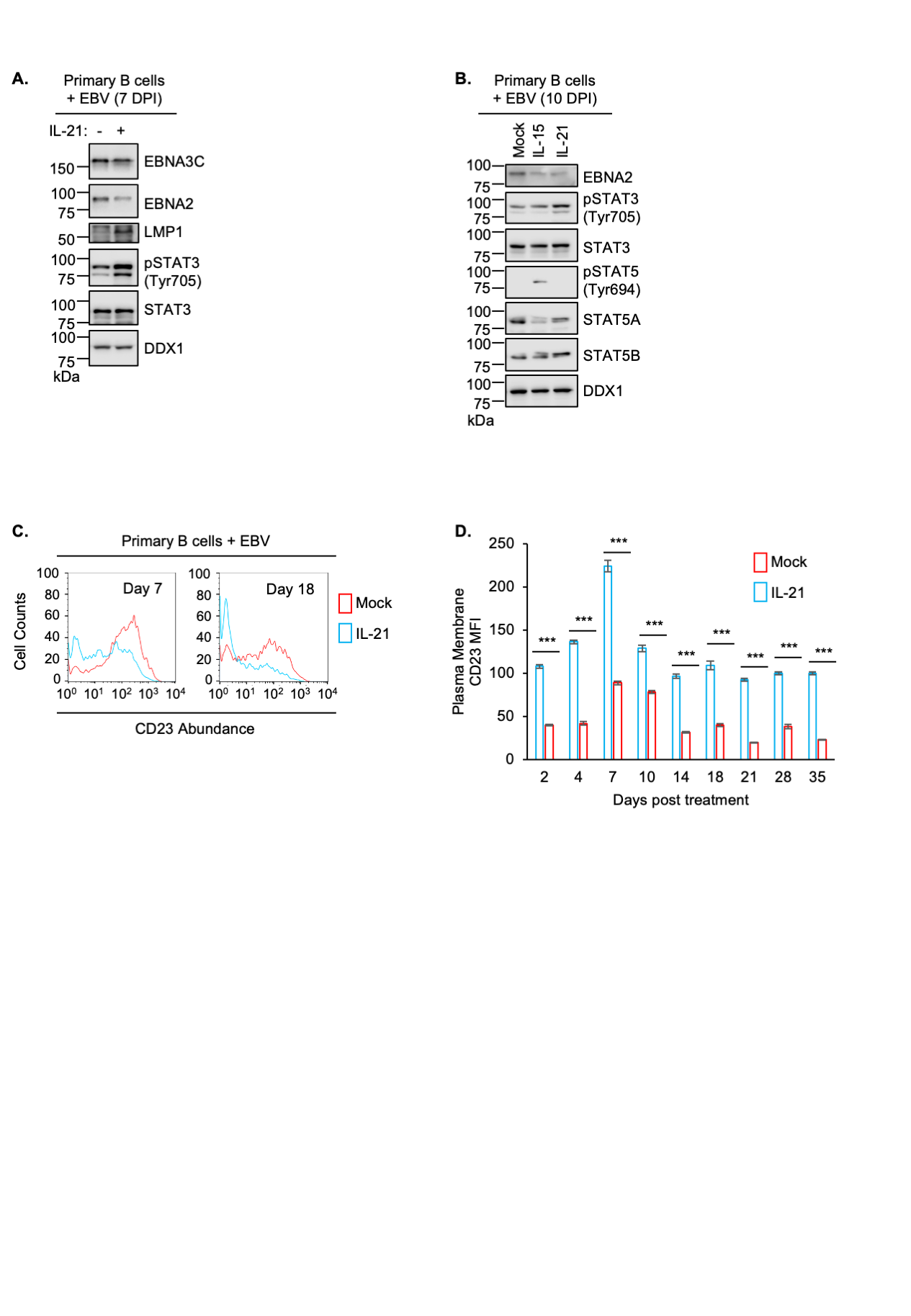

Supplement: S9 Fig — (A) Immunoblot analysis of WCL from primary human B cells at 7 DPI, which were then mock treated or stimulated with IL-21 for six days. (B) Immunoblot analysis of WCL from primary B cells at 10 DPI, mock treated or treated with IL-15 or IL-21 for six days. (C) Plasma membrane CD23 abundances in primary human B-cell mock treated or treated with IL-21 (100 ng/ml) at Day 7 vs 18 post-infection by Akata EBV. IL-21 was refreshed every 2 days. (D) Mean ± SD CD23 abundances from n = 3 replicates of primary B-cells infected by Akata EBV in the absence or presence of IL-21, as in (C), ***p < 0.001. Blots are representative of n = 3 replicates. (TIFF) [file ppat.1011939.s009.tiff]
